# Therapeutic targeting of Lyn kinase to treat chorea-acanthocytosis

**DOI:** 10.1186/s40478-021-01181-y

**Published:** 2021-05-03

**Authors:** Kevin Peikert, Enrica Federti, Alessandro Matte, Gabriela Constantin, Enrica Caterina Pietronigro, Paolo Francesco Fabene, Paola Defilippi, Emilia Turco, Federico Del Gallo, Pietro Pucci, Angela Amoresano, Anna Illiano, Flora Cozzolino, Maria Monti, Francesca Garello, Enzo Terreno, Seth Leo Alper, Hannes Glaß, Lisann Pelzl, Katja Akgün, Tjalf Ziemssen, Rainer Ordemann, Florian Lang, Anna Maria Brunati, Elena Tibaldi, Immacolata Andolfo, Achille Iolascon, Giuseppe Bertini, Mario Buffelli, Carlo Zancanaro, Erika Lorenzetto, Angela Siciliano, Massimiliano Bonifacio, Adrian Danek, Ruth Helen Walker, Andreas Hermann, Lucia De Franceschi

**Affiliations:** 1 Translational Neurodegeneration Section “Albrecht-Kossel”, Department of Neurology, University Medical Center Rostock, University of Rostock, Gehlsheimer Straße 20, 18147 Rostock, Germany; 2 Department of Medicine, University of Verona, Policlinico GB Rossi, P.Le L. Scuro, 10, 37134 Verona, Italy; 3 Department of Neuroscience, Biomedicine and Movement Sciences, University of Verona, Verona, Italy; 4 Department of Biotecnologie Molecolari E Scienze Per La Salute, University of Torino, Turin, Italy; 5 Department of Chemical Sciences, University Federico II of Napoli, Naples, Italy; 6 Molecular Imaging Center - Department of Molecular Biotechnology and Health Sciences, University of Torino, Turin, Italy; 7 Division of Nephrology, Beth Israel Deaconess Medical Center, Harvard Medical School, Boston, MA USA; 8 Department of Physiology I, Eberhard Karl University, Tübingen, Germany; 9 Transfusion Medicine, Eberhard Karl University, Tübingen, Germany; 10 Department of Neurology, University Hospital Carl Gustav Carus, Technische Universität Dresden, Dresden, Germany; 11 Medical Department I, University Hospital Carl Gustav Carus, Technische Universität Dresden, Dresden, Germany; 12 Department of Molecular Medicine, University of Padua, Padua, Italy; 13 Department of Molecular Medicine and Medical Biotechnology, University of Naples Federico II, Naples, Italy; 14 Department of Neurology, James J. Peters Veterans Affairs Medical Center, Bronx, NY USA; 15 Department of Neurology, Ludwig Maximilians University of Munich, Munich, Germany; 16 Department of Neurology, Icahn School of Medicine at Mount Sinai, New York, NY USA; 17 Centro Piattaforme Tecnologiche, University of Verona, Verona, Italy; 18 Center for Transdisciplinary Neurosciences Rostock (CTNR), University Medical Center Rostock, University of Rostock, Rostock, Germany; 19 Division for Neurodegenerative Diseases, Department of Neurology, Technische Universität Dresden, Dresden, Germany; 20 Center for Regenerative Therapies Dresden, Dresden, Germany; 21 CEINGE Biotecnologie Avanzate, Naples, Italy

**Keywords:** Chorein, Lyn, Cell signaling, Basal ganglia, Neurodegeneration

## Abstract

Chorea-Acanthocytosis (ChAc) is a devastating, little understood, and currently untreatable neurodegenerative disease caused by *VPS13A* mutations. Based on our recent demonstration that accumulation of activated Lyn tyrosine kinase is a key pathophysiological event in human ChAc cells, we took advantage of *Vps13a*^*−/−*^ mice, which phenocopied human ChAc. Using proteomic approach, we found accumulation of active Lyn, γ-synuclein and phospho-tau proteins in *Vps13a*^*−/−*^ basal ganglia secondary to impaired autophagy leading to neuroinflammation. Mice double knockout *Vps13a*^*−/−*^ Lyn^−/−^ showed normalization of red cell morphology and improvement of autophagy in basal ganglia. We then in vivo tested pharmacologic inhibitors of Lyn: dasatinib and nilotinib. Dasatinib failed to cross the mouse brain blood barrier (BBB), but the more specific Lyn kinase inhibitor nilotinib, crosses the BBB. Nilotinib ameliorates both *Vps13a*^*−/−*^ hematological and neurological phenotypes, improving autophagy and preventing neuroinflammation. Our data support the proposal to repurpose nilotinib as new therapeutic option for ChAc patients.

**Supplementary Information:**

The online version contains supplementary material available at 10.1186/s40478-021-01181-y.

## Introduction

The ultra-rare neurodegenerative disease Chorea-Acanthocytosis (ChAc) with 1000–5000 cases worldwide is one of the core neuroacanthocytosis syndromes (NA) [26, 50, 70]. NA diseases manifest with a spectrum of neurological symptoms in addition to the presence of misshaped red blood cells (RBCs) with thornlike protrusions, referred to as acanthocytes [7, 26, 35, 50]. Autosomal-recessive ChAc is caused by loss-of-function mutations in the *vacuolar protein sorting 13 homolog A* (*VPS13A*) gene encoding the chorein polypeptide gene product [8, 10, 54, 68]. Only symptomatic treatment is currently available to modify the devastating disease course [71] despite a shortened life-span marked by considerable morbidity and compromised independent living. These clinical manifestations are accompanied by loss of striatal medium spiny neurons [38] and a distinctive cortical neurodegeneration [39]. Other members of the vacuolar protein sorting (Vps) family of proteins have been linked to more common neurodegenerative disorders such as Parkinson’s disease (PD) (Vps35 and Vps13c) and frontotemporal dementia (Vps4B) [34, 60].

ChAc patients often present with progressive movement disorders like chorea, parkinsonism and/or dystonia [13, 53, 70]. Therefore, ChAc should be considered as a relevant differential diagnosis of Huntington’s disease (HD) [22]. This disease severely affects independent living, and results in significant morbidity and a markedly reduced life-span [71].

*Although* studies in mammalian cell lines and in model organisms such as yeast suggest that chorein’s possible function as a lipid transporter at organelle contact sites, possibly mediating non-vesicular phospholipid transport [14, 32, 74], the function of chorein remains incompletely understood. We recently identified accumulation of active Lyn, a kinase of the Src family tyrosine kinases as key driver of ChAc pathophysiology (for review see [50]). We also found that Lyn inhibition by PP2 or dasatinib (1) ameliorates the distorted erythroid morphology and other altered red cell features in vitro; and (2) ameliorates the pathologically increased synaptic transmission in striatal medium spiny neurons generated from ChAc iPSCs [9, 42, 64]. The proteotoxic effect of Lyn gain-of-function reflects impaired autophagy, in agreement with VPS13A-deficient cell models.

Here, we studied Vps13a^−/−^ mice lacking chorein. Vps13a^−/−^ mice display biological features of human ChAc. We confirmed the accumulation of active Lyn in both RBCs and basal ganglia of Vps13a^−/−^ mice, associated with impaired autophagy and accumulation of phospho-Tau proteins and γ-synuclein. Dasatinib treatment of Vps13a^−/−^ mice blocked Lyn activity in RBCs but not in the basal ganglia. Mass spectrometric analysis revealed that dasatinib did not accumulate to detectable levels in basal ganglia, thus providing one potential explanation for the absence of an effect of dasatinib on neurologic phenotype of ChAc patients. We therefore tested the second generation TKI nilotinib, with higher selectivity for Lyn and able to cross the blood–brain barrier (BBB) [21]. We showed that nilotinib reached the basal ganglia, where it inhibited Lyn and improved autophagy, neuronal loss and neuroinflammation.

## Materials and methods

### Mouse model for ChAc (Vps13a^−/−^ mice)

Experiments were performed on age and gender- matched WT (C57BL/6J) and Vps13a^−/−^ mice and on 12 months-old sex-matched Vps13a^−/−^Lyn^−/−^ mice. We obtained Vps13a heterozygous knock out (±) mice from the EMMA Consortium (Additional file 1: Fig. S1). Vps13a^−/−^Lyn^−/−^ mice were generated in house backcrossing for at least 16 generation Vps13a^−/−^ mice and Lyn^−/−^ mice. The Institutional Animal Experimental Committee of University of Verona (CIRSAL) approved the experimental protocol. Whenever indicated WT and Vps13a^−/−^ mice were daily treated with vehicle (tap water) or dasatinib or nilotinib. Dasatinib (50 mg/Kg) was administered by daily gavage to 12 months-old mice for 1 month. Nilotinib (25 mg/Kg/day) was administered by gavage to 11 months-old Vps13a^−/−^ mice for either 6 weeks or 3 months or 6 months. This dosage was chosen based on a previous report on a mouse model for PD and AD [20, 40]. Isoflurane-anesthetized mice were randomly assigned to experimental groups and blindly analyzed. Hematologic parameters and red cell indices were evaluated on a Siemens ADVIA 2120 hematology analyzer. Hematocrit and hemoglobin were manually determined [27, 43, 44]. Acanthocyte evaluation and counting were performed on McGrawald-Giemsa-stained blood smears and by electron microscopy [42]. Brains immediately removed from euthanized mice were dissected to isolate basal ganglia (BG) and cortex tissues, which were instantly frozen in liquid nitrogen.

### Statistical analysis

Statistical analysis was performed with the GraphPad Prism 8.0 program. Data were analyzed using either *t*-test or one-way ANOVA (Dunnet’s test) for longitudinal studies or 2-way analysis of variance (ANOVA) with Bonferroni connection for repeated measures among mice of different genotypes. *p* < 0.05 was considered significant.

#### Behaviour test

Comparisons between two groups were performed using the two-tailed unpaired Student’s *t*-test. Data were expressed as the mean ± SEM. Statistical significance was accepted at the 95% confidence level (*p* < 0.05). Spontaneous locomotor activity was evaluated using a two-way mixed-model ANOVA (strain*day) followed by the Bonferroni post hoc test. For the analysis of gait parameters, the means of the hind and front paws and individual paws were considered. The individual averages for each mouse were calculated over three good runs (straightforward and without hesitations), and the differences between groups were evaluated with a two-tailed unpaired Student’s *t*-test. Statistical significance was accepted at the 95% confidence level (*p* < 0.05).

#### Immunofluorescence microscopy

Quantification of NeuN positive cells and Iba1 positive cells was followed by statistical analyses applying two-tailed Unpaired t test. Data are shown as mean ± SEM. Quantification of beclin1-positive and γ-Synuclein-positive cells were followed by a statistical analysis. Statistically significant differences between the two non-parametric data sets were assessed by Mann–Whitney’s test. Statistical significance was determined at *p* < 0.05.

#### Brain spectroscopy

Comparisons between two groups were performed using the two-tailed unpaired Student’s *t*-test. Data were expressed as the mean ± SEM. Statistical significance was accepted at the 95% confidence level (*P* < 0.05).

#### NanoLC/MS–MS analysis

Statistical analysis was performed in MeV using a Student’s two tailed t-test. Statistical significance was determined at *P* < 0.05.

## Results

### Vps13a^−/−^ mice recapitulate key features of patients suffering from ChAc

Survival of Vps13a^−/−^ mice was significantly reduced compared to wild-type animals as assessed by log-rank test analysis (Additional file 1: Fig. S1.2A). Weights of both male and female *Vps13a*^*−/−*^ mice of across all ages were lower than those of wild-type animals (Additional file 1: Fig. S1.2B).

#### Hematologic phenotype

**Fig. 1:**
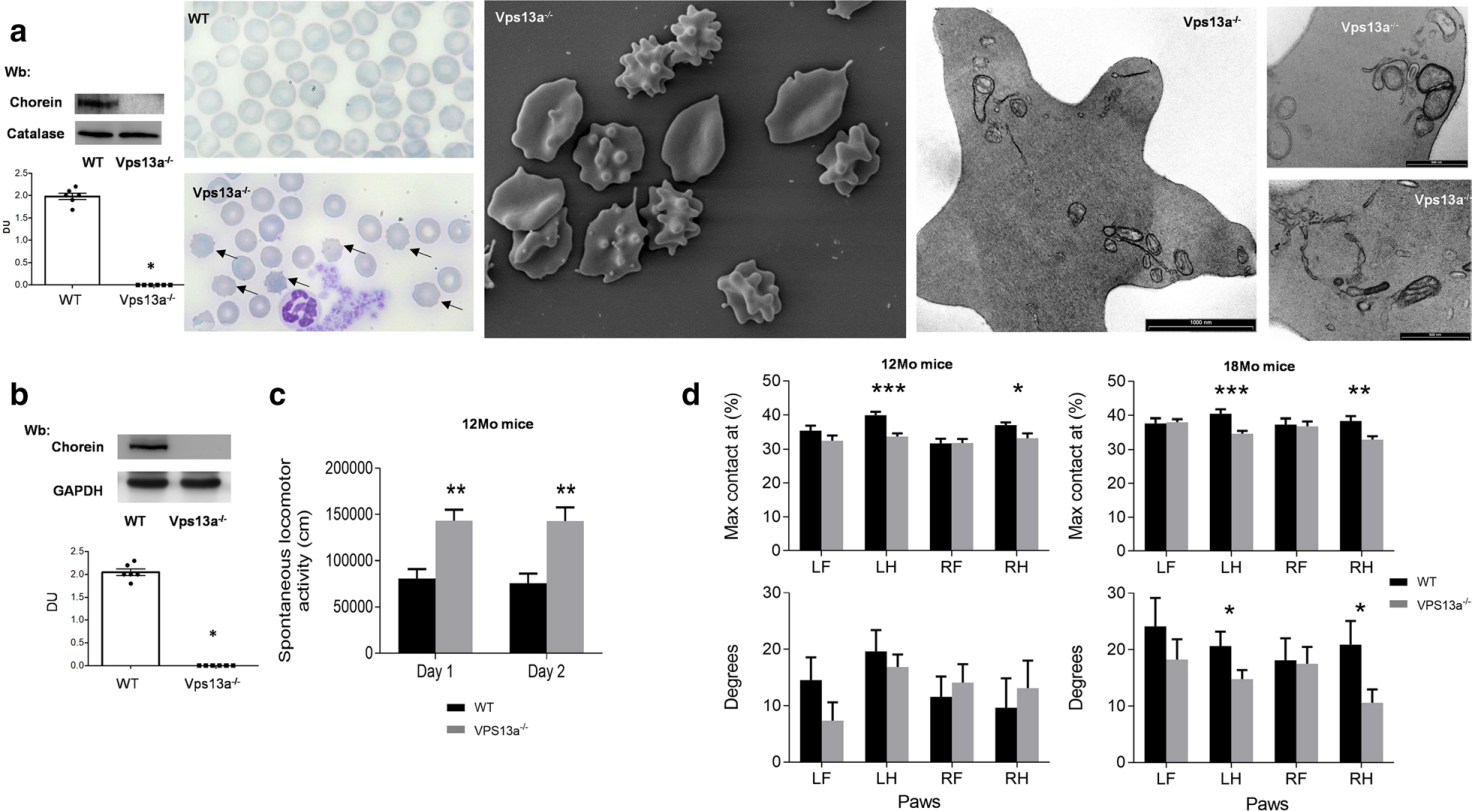
*Vps13a*^*−/−*^ mice exhibit hematologic and neurologic features similar to those in human ChAc. **a** Left panel. Western blot (Wb) analysis of chorein in red cells of WT and Vps13a^−/−^ mice. Catalase served as protein loading control. Lower panel. Densitometric analysis (arbitrary units) of immunoblot bands like those shown; means ± SEM (n = 6; *p < 0.001 vs. WT by t-test). Right panel (from left to right). Morphologic analysis of peripheral blood from wild-type (WT) and Vps13a^−/−^ mice. Blood smears stained with May-Grunwald-Giemsa. Cells were imaged under oil at 100 × magnification using Panfluor objective 1.30 numeric aperture on a Nikon Eclipse DS-5 M camera and processed with Digital Slide (DS-L1) Nikon. Black arrows indicate acanthocytes in Vps13a^−/−^ mice. (see also Additional file 1: Fig. S4.2C). Electron microscopy of circulating red cells from Vps13a^−/−^ mice. The image is representative of 10 similar imaged visual fields for each of 10 Vps13a^−/−^ mice at the ages of 12 months. **b** Western blot (Wb) analysis of Chorein in isolated basal ganglia of wild-type (WT) and Vps13a^−/−^ mice. GADPH is the loading control. Densitometric analysis (arbitrary units) of the immunoblot bands similar to those shown are presented (bottom); data are means ± SEM (n = 6; *P < 0.001 vs. WT by t-test). **c** Spontaneous locomotor activity in Vps13a^−/−^ and wild-type mice in undisturbed conditions. At 12 months mice were maintained in a PhenoTyper® cage (Noldus®) and continuously monitored for two consecutive days (Day 1 and Day 2). Data represent the mean ± SEM of the total distance moved (cm) *per* day (**P < 0.01 vs. wild-type mice, n = 6 animals *per* group). **d** CatWalk® gait analysis of Vps13a^−/−^ and wild-type mice. The data represent the mean ± SEM of three runs *per* animal and are presented *per* each paw, left front (LF), left hind (LH), right front (RF) and right hind (RH) (*p < 0.05, **P < 0.01, ***P < 0.001; n: 19 Vps13a^−/−^ mice, n: 15 wild-type mice) (see also Additional file 1: Fig. S5A). At 18 months of age Vps13a^−/−^ mice showed a deviating paw angle of both hind paws compared to controls *P < 0.05; n = 19 Vps13a^−/−^ mice, n = 15 wild-type mice)

Chorein expression was undetectable in Vps13a^−/−^ mouse RBCs (Fig. 1a). Acanthocytes were observed by multiple imaging approaches (Fig. 1a). The numbers of acanthocytes in *Vps13a*^*−/−*^ mice were stable beyond age 2 months, considered as adult subjects from the perspective of hematologic development (Additional file 1: Fig. S1.2C). No major differences in hematologic parameters or red cell indices were observed in Vps13a^−/−^ mice compared to wild-type animals with the exception of the Hb distribution width (HDW), useful to evaluate acanthocytes (Table 1). HDW was significantly increased in Vps13a^−/−^ mice compared to wild-type animals (Table 1). We fractionated RBCs as a function of cell Hb content and cell volume (V/HC), revealing a dense cell fraction only in Vps13a^−/−^ mice (Additional file 1: Fig. S1.2C, lower panel, blue circle). This fraction contains acanthocytes similarly to those observed in human ChAc [42]. As like human ChAc, osmotic fragility of Vps13a^−/−^ mouse RBCs was higher than in wild-type RBCs (Additional file 1: Fig. S1.2D). This was associated with reduced K^+^ content in *Vps13a*^*−/−*^ RBCs as compared to healthy mouse RBCs (WT mice 460 ± 12 mmol/Kg Hb vs. *Vps13a*^*−/−*^ mice 350 ± 8.1 mmol/Kg Hb *n* = 6 in each group, *p < 0.05), resembling again the human disease [5].

**Table 1:** Hematological parameters and red cell indices in wild-type and Vps13a^−/−^ mice

	Wild-type mice (*n* = 12)	Vps13a^−/−^ mice (*n* = 12)
Hct (%)	45.3 ± 1.2	46.1 ± 0.8
Hb (g/dl)	14.9 ± 0.3	14.2 ± 0.6
MCV (fl)	52.4 ± 1.6	53.1 ± 1.5
MCH (pg)	16.3 ± 0.8	15.9 ± 0.8
CHCM (g/dL)	26.3 ± 0.2	26.2 ± 0.2
RDW (%)	12.5 ± 0.4	12.9 ± 0.7
HDW (g/dL)	2.1 ± 0.43	3.1 ± 0.47*
Retics (cell/uL)	417 ± 65	382 ± 84

*Hct* haematocrit, *Hb* haemoglobin, *MCV* mean corpuscular volume, *MCH* mean corpuscular haemoglobin, *CHCM* cell hemoglobin mean content, *RDW* red cell distribution width, *HDW* hemoglobin distribution width, *Retics* reticulocytes *p < 0.05 compared to wild-type mice

In Vps13a^−/−^ mouse RBCs, we found accumulation of active Lyn compared to wild-type erythrocytes (Additional file 1: Fig. S1.2E). This increase was associated with retention of double membrane remnants and vesicles, indicating an impairment of autophagy (Fig. 1a). In agreement with the morphological findings, we observed accumulation of Ulk1, Atg4, Atg5 and Rab5 polypeptides, as seen in human ChAc RBCs [42] (Additional file 1: Fig. S1.2F). Taken together these data recapitulate key hematologic features of ChAc patients.

#### Neurologic phenotype

Chorein was undetectable in isolated basal ganglia from saline buffer-perfused 12- and 18-months old *Vps13a*^*−/−*^ mice (Fig. 1b). Gait and motor assessment were performed in cohorts of Vps13a^−/−^ (n:19) and wild-type (n:15) mice at 12 and 18 months. Anxiety trait and spontaneous locomotor activity were assessed in a subgroup (n:6 for each strain) of both mouse strains (12 months-old only) applying a 5-min protocol using elevated-plus maze (EPM). No significant differences were found in the time spent in the closed and open arms (an index related to a more or a less anxiety trait) between Vps13a^−/−^ and wild-type mice. The spontaneous locomotor activity was continuously monitored for two days by video-tracking observations of individual 12 months-old mice in PhenoTyper cages, which allowed home-cage evaluation of locomotor activity, unaffected by anxiety and/or stress of a test cage environment. The overall distance covered by the Vps13a^−/−^ mice was significantly higher (day 1: 82.48 ± 11.68%, *p* < 0.01; day 2: 100.29 ± 11.68%, *p* < 0.01) compared to that of age-matched wild-type mice (Fig. 1c). Vps13a^−/−^ mice and matched wild-type mice were tested for their spontaneous gait behavior (Fig. 1d). The gait performance of Vps13a^−/−^ mice was affected during the test session at both 12 and 18 months of age. In particular, starting from 12 months, Vps13a^−/−^ mice showed a longer duration from the start of a run until maximum contact occurs for both hind paws compared to age- and weight-matched control mice (see also Additional file 1: Fig. S2A for schematic diagram of the angle evaluation). The delay in reaching the maximum contact of paws with a glass plate was also observed at older age (Fig. 1d). Also, at 18 months, but not at 12 months (Fig. 1d), Vps13a^−/−^ mice showed a different paw angle of both hind paws (Fig. 1d). A similar discrepancy in paw angle was reported in a transgenic rat model for Huntington’s disease [69]. “Max contact at (%)” refers to the duration, from the start of a run, until maximum contact of paws occurs. This index has been largely studied in rodent models in the context of parkinsonism [2, 3]. The basal ganglia of these aged *Vps13a*^*−/−*^ mice exhibited accumulation of active Lyn as compared to wild-type animals (Additional file 1: Fig. S2B, C), in agreement with our previous report on active Lyn accumulation in neuronal cells derived from ChAc iPSC [64]. We note, in particular the presence in *Vps13a*^*−/−*^ mouse basal ganglia of active Lyn stabilized in high molecular weight complexes, as earlier observed in human ChAc RBCs (Additional file 1: Fig. S2D) [42]. Collectively, our data indicate that *Vps13a*^*−/−*^ mice recapitulate biological features and neurological phenotype like those of ChAc patients.

### Vps13a^−/−^ mice show neuronal loss and neuroinflammation

**Fig. 2:**
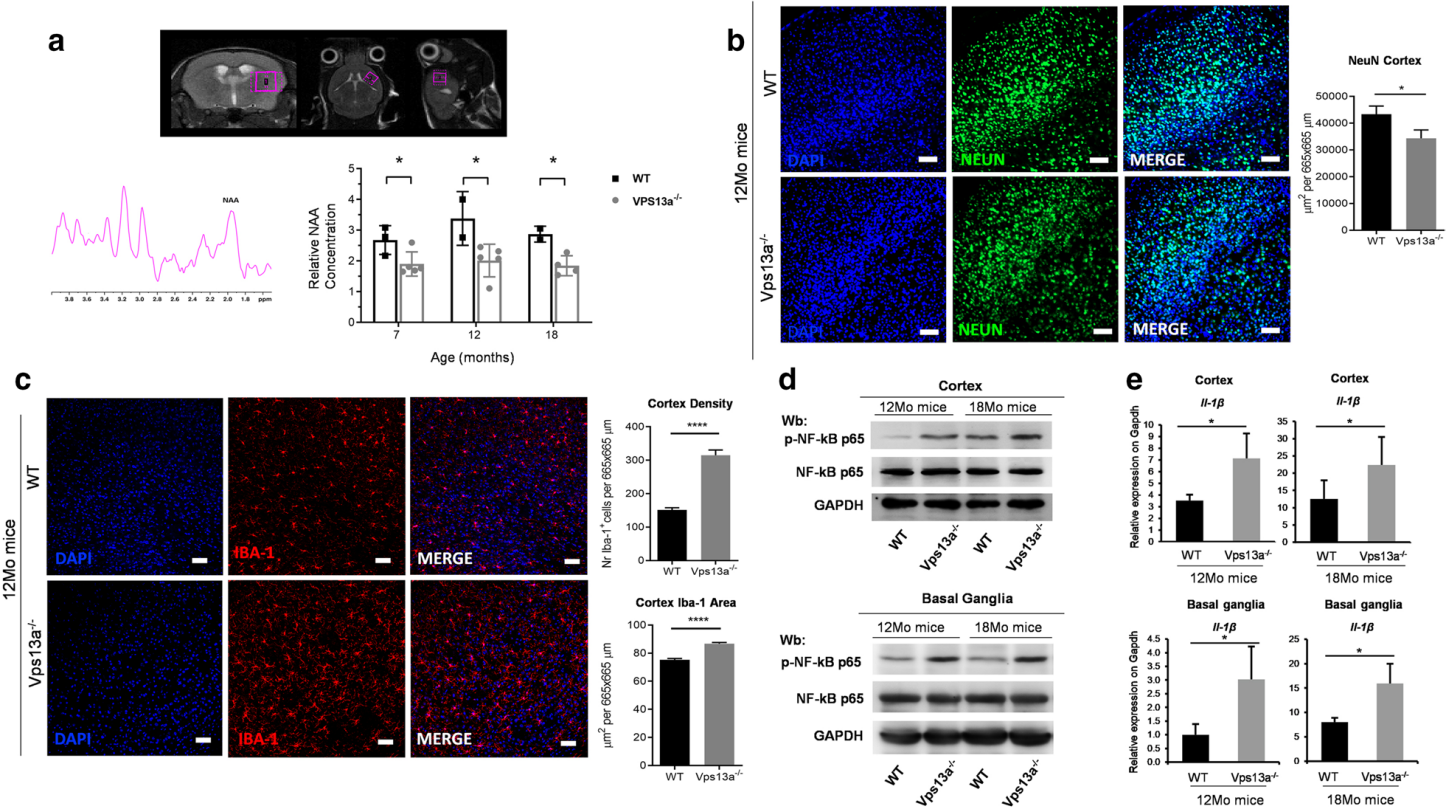
Vps13a^−/−^ mice show neuronal loss associated with signs of neuroinflammation*.* **a** N-Acetyl Aspartate (NAA) concentration determined by 1H MRS (7 T) in the striatum of Vps13a^−/−^ or WT control mice at different age (7, 12 and 18 months). NAA concentration was normalized using creatine as internal reference. Data are mean ± SEM (*p < 0.05 by t-test vs. WT). **b** Representative images of NeuN staining in cortex of WT control and Vps13a^−/−^ mice at 12 months of age. Neurons in green, nuclei in blue. Scale bar: 50 mm (Objective 20x). Quantification of NeuN-positive cells area in cortex. Results are expressed as mean ± SEM. **c** Representative images of Iba-1 positive microglia cells in cortex of WT control and Vps13a^−/−^ mice at 12 months of age (Microglia in red, nuclei in blue). Scale bar:50 mm. Quantitative analysis of microglia show significant differences in microglial density and activation in the cortex of Vps13a^−/−^ compared to WT control mice. Results are expressed as mean ± SEM (****P < 0.0001; Unpaired t-test) **d** Western blot (Wb) analysis of phospho-NF-kB and total NF-kB in isolated basal ganglia of wild-type (WT) and *Vps13a*^*−/−*^ mice at 12 and 18 months (Mo) of age. GADPH is the loading control. Densitometric analysis is shown in Additional file 1: Fig. S6. **e** mRNA expression of interleukine-1β (Il-1b) by qRT-PCR on cortex and basal ganglia from 12 and 18 months (Mo) old WT and *Vps13a*^*−/−*^ mice. Experiments were performed in triplicate. Data are mean ± SD. *P < 0.05 compared with WT mice using ANOVA; internal comparisons were calculated by unpaired student t-test

To explore neurochemical abnormalities in striatum from Vps13a^−/−^ mice by non-invasive approaches, we used proton magnetic resonance spectroscopy H-MRS [1, 11, 31]. We found a significant reduction in N-acetylasparate (NAA) in striatum from Vps13a^−/−^ mice compared to wild-type animals, suggesting the presence of neuronal degeneration in mice genetically lacking Chorein (Fig. 2a) [11, 51]. Histopathological studies of Vps13a^−/−^ mice demonstrated a significant reduction in NeuN staining in cortex compared to wild-type animals, indicating a loss of neurons (Fig. 2b).

It is noteworthy that we found increased microglia in the cortex from 12 months-old Vps13a^−/−^ mice compared to wild-type animals (Fig. 2c). This was associated with increased activation of NF-kB p65 in both cortex and basal ganglia from 12 and 18 months-old Vps13a^−/−^ mice compared to wild-type animals, consistent with neuroinflammation in mice genetically lacking Chorein (Fig. 2d, see also Additional file 1: Fig. S3). In agreement with this observation, we noted up-regulation of IL-1β mRNA expression in both cortex and basal ganglia from 12 and 18 months-old Vps13a^−/−^ mice compared to wild-type animals (Fig. 2e).

Taken together, our data indicate neuron loss and neuro-inflammation in *Vps13a*^*−/−*^ mice, highlighting similarities with other neurodegenerative diseases such as Parkinson disease [23].

### Vps13a^−/−^ mice show impaired autophagy involving beclin-1 pathway

To understand the possible contribution of impaired autophagy to neuronal dysfunction and neuroinflammation in Vps13a^−/−^ mice, we evaluated expression of key autophagy flux proteins in isolated basal ganglia from saline buffer-perfused Vps13a^−/−^ and wild-type mice. *Vps13a*^*−/−*^ basal ganglia exhibited activation of LC3 II/I and significant accumulation of the following autophagy-related proteins: Ulk1, Atg4, Atg5, Atg9 and the lysosomal cargo protein p62 consistent with impairment of autophagy in Vps13a^−/−^ mice (Additional file 1: Fig. S4A) as described in other neurodegenerative diseases such as PD and AD [45, 56–58]. The beclin-1 system is one of the most critical hubs of the autophagic process, playing an important role in initiation and promotion of autophagy. Severe neurodegeneration and impaired autophagy have been observed in beclin1^−/−^ mice, further linking beclin-1-dependent autophagy with neurodegenerative diseases [45, 58]. In Vps13a^−/−^ mice at 12 and 18 months of age, beclin-1 was significantly reduced as observed by different methods (Additional file 1: Fig. S4B, C). We also observed an accumulation of the beclin-1 complex components Vps34 and Rab5 as well as of p62, a marker of late phase of autophagy (Additional file 1: Fig. S5A). To further evaluate a possible link between chorein and beclin-1, we immunoprecipitated Beclin-1 and immunoblotted for either Chorein, Atg14L or Vps34. We found Chorein co-immunoprecipitated with Beclin-1 only in basal ganglia from wild-type mice but not from *Vps13a*^*−/−*^ mice. In addition, we observed a reduction in Vps34 and Atg14L association with beclin-1 in *Vps13a*^*−/−*^ mice compared to wild-type animals (Additional file 1: Fig. S5B). Noteworthy, we found up-regulation of beclin-1 mRNA levels in isolated basal ganglia from Vps13a^−/−^ mice compared to wild-type mice, suggesting a Beclin-1 protein degradation (Additional file 1: Fig. S5C). Since Beclin-1 levels depend on Caspase 3 activity, we evaluated Caspase-3 activation by immunoblot analysis of total cleaved Caspase-3 and a fluorometric assay for Caspase-3 activity. In basal ganglia of Vps13a^−/−^ mice, Caspase-3 activity was increased compared to wild-type animals (Additional file 1: Fig. S5D). It is of note that increased Caspase-3 activity has been also reported in brains from PD patients [4, 19]. In Vps13a^−/−^ mice, the perturbation of autophagy resulted in accumulation of polyubiquitinated proteins in basal ganglia compared to wild-type animals, further supporting the impairment of autophagy in Vps13a^−/−^ mice (Additional file 1: Fig. S5E).

### Vps13a^−/−^Lyn^−/−^ mice show reduced acanthocytes, amelioration of autophagy and decreased activity of NF-kB p65, linked to neuroinflammation

**Fig. 3:**
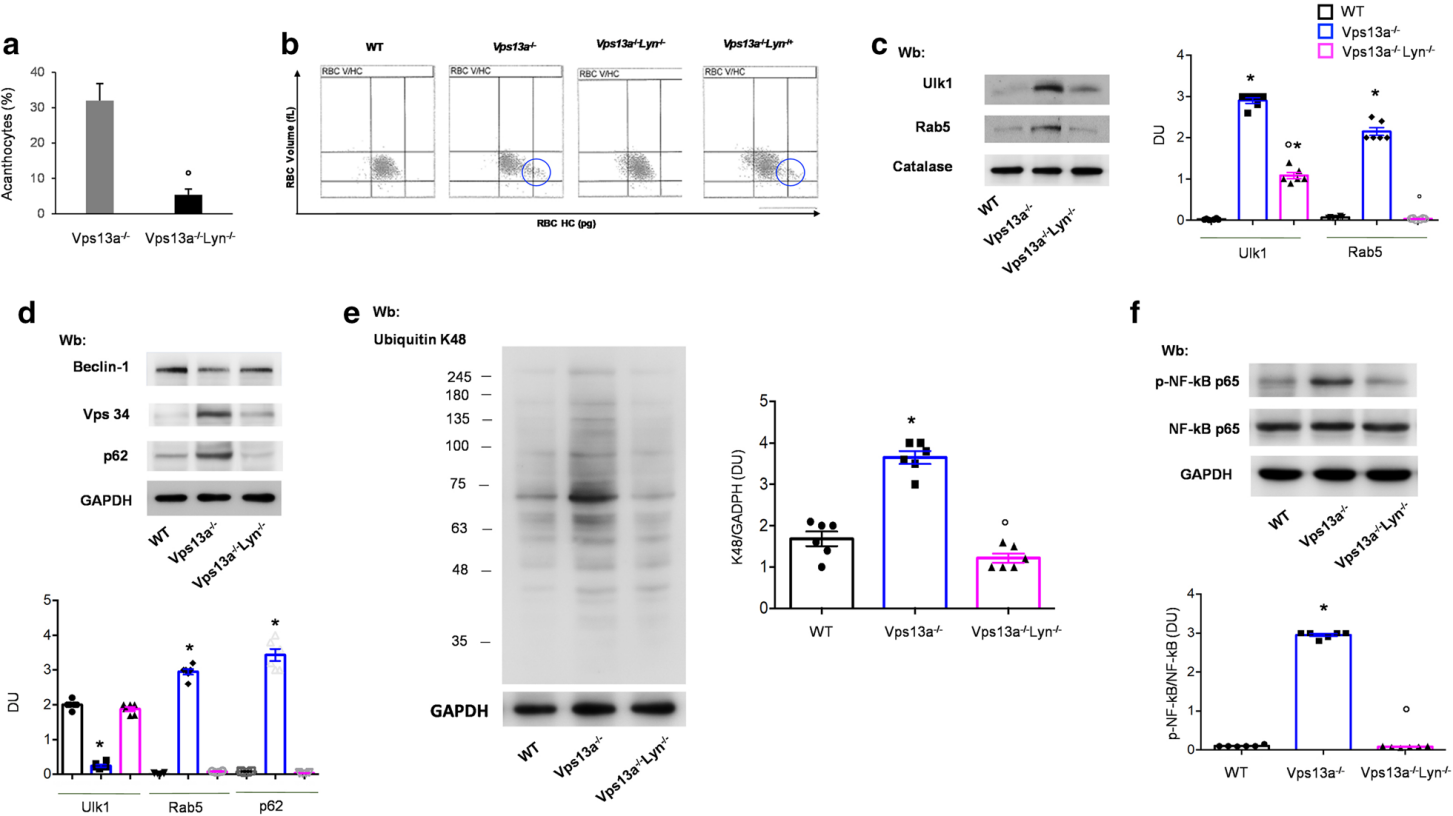
Vps13^−/−^Lyn^−/−^ mice show amelioration of hematologic phenotype and improvement of autophagy and neuroinflammation in basal ganglia. **a** Quantitation of acanthocytes by brightfield microscopic analysis of peripheral blood smears from *Vps13a*^*−/−*^ and *Vps13a*^*−/−*^*Lyn*^*−/−*^ mice. Data from 50 visual fields was collected by two blinded researchers. Results are means ± SEM n = 6; °*p* < 0.002 versus *Vps13a*^*−/−*^ by t-test **b** Left panel. Red cell distribution histograms generated for red blood cell volume (RBC Volume) and cell haemoglobin concentration (RBC-HC) of RBCs from wild-type (WT) control, *Vps13a*^−/−^, *Vps13a*^*−/−*^*Lyn*^*−/−*^ and *Vps13a*^*−/−*^*Lyn*^*−/*+^ mice. One representative experiment of six with similar results is shown.The blue circle indicates the presence of a subpopulation of dense red cells containing acanthocytes, as described in human patients (Lupo et al. [42]). **c** Western blot (Wb) analysis of Ulk1 (Atg1) and Rab 5 from red cell cytosolic fractions of wild-type (WT), *Vps13a*^*−/−*^ and *Vps13a*^*−/−*^*Lyn*^*−/−*^ mice. Catalase was used as protein loading control. Densitometric analyses of the immunoblot bands similar to those shown are presented at right. Data are means ± SEM (n = 6; *P < 0.02 vs. WT; °*P* < 0.05 vs. *Vps13a*^*−/−*^ by two-way-ANOVA/Bonferroni’s multiple comparison test). **d** Western blot (Wb) analysis of Beclin-1, Vps34 and p62 in isolated basal ganglia of wild-type (WT), *Vps13a*^*−/−*^ and *Vps13a*^*−/−*^*Lyn*^*−/−*^ mice. GAPDH served as protein loading control. Densitometric analyses of the immunoblot bands similar to those shown are presented at right. Data are means ± SEM (n = 6; *p < 0.02 vs. WT; °*p* < 0.02 compared to *Vps13a*^*−/−*^ mice by two-way-ANOVA/Bonferroni’s multiple comparison test). **e** Western blot (Wb) analysis of K48-ubiquitinated proteins in basal ganglia isolated from wild-type (WT), *Vps13a*^*−/−*^ and *Vps13a*^*−/−*^*Lyn*^*−/−*^ mice. GAPDH served as protein loading control. Densitometric analyses of the immunoblot bands similar to those shown are presented at right. Data are means ± SEM (n = 6; *p < 0.02 vs. WT; °*p* < 0.02 compared to Vps13a^−/−^ mice by two-way-ANOVA/Bonferroni’s multiple comparison test). **f** Western blot (Wb) analysis of phospho-NF-kB p65 (P-NF-kB), NF-kB in isolated basal ganglia of wild-type (WT), *Vps13a*^*−/−*^ and *Vps13a*^*−/−*^*Lyn*^*−/−*^ mice. GAPDH served as protein loading control. Densitometric analyses of the immunoblot bands similar to those shown are presented at right. Data are means ± SEM (n = 6; *p < 0.02 vs. WT; °*p* < 0.02 compared to *Vps13a*^*−/−*^ mice by two-way-ANOVA/Bonferroni’s multiple comparison test)

To further explore the role of Lyn in Vps13a^−/−^ mice, we generated a Vps13a^−/−^Lyn^−/−^ double knockout mouse (Fig. 3 and Additional file 1: Fig. S6, S7). Since Lyn^−/−^ mice have been previously characterized and little role for Lyn kinase has been documented on primary neuronal functions [17, 18], we focused on the comparison between Vps13a^−/−^ and Vps13a^−/−^Lyn^−/−^ mice. In addition, the hematologic phenotype of *Lyn*^*−/−*^ mice of age less than 15 months is similar to that of wild-type animals (Table S2) [25, 29]. In 12 months-old Vps13a^−/−^ Lyn^−/−^ mice, we observed significantly reduced acanthocyte numbers compared to either *Vps13a*^*−/−*^ or to *Vps13a*^*−/−*^*Lyn*^*−/*+^ mice. This finding was accompanied by (1) disappearance of the dense red cell fraction; (2) the reduction in HDW and (3) the reduction of red cell osmotic fragility in Vps13a^−/−^ Lyn^−/−^mice compared to both Vps13a^−/−^ and Vps13a^−/−^ Lyn^−/+^ mice (Fig. 3a, b, Additional file 1: Fig. S6A-B). *Vps13a*^*−/−*^ mice genetically deficient in Lyn also exhibited normalization of the red cell content of autophagy-related proteins as compared to their elevated contents in *Vps13a*^*−/−*^ mouse erythrocytes (Fig. 3c). In isolated basal ganglia from Vps13a^−/−^ Lyn^−/−^ mice, we found a slight but significant increase in beclin-1 and a marked reduction in accumulation of Vps34 and p62, supporting the dysregulated autophagy in mice lacking chorein by the incremental absence of Lyn (Fig. 3d). Indeed, we further observed significantly reduced accumulation of polyubiquitinated proteins in the basal ganglia from Vps13a^−/−^ Lyn^−/−^ mice compared to Vps13a^−/−^ animals (Fig. 3e). This was associated with reduced NF-kB p65 activation, suggesting a reduction in neuroinflammation in Vps13a^−/−^ Lyn^−/−^ mice (Fig. 3f). Collectively these data support a key role of Lyn in disease mechanism of ChAc.

### Proteomic analyses of Vps13a^−/−^ mouse basal ganglia revealed abnormal proteostasis and accumulation of γ-synuclein

**Fig. 4:**
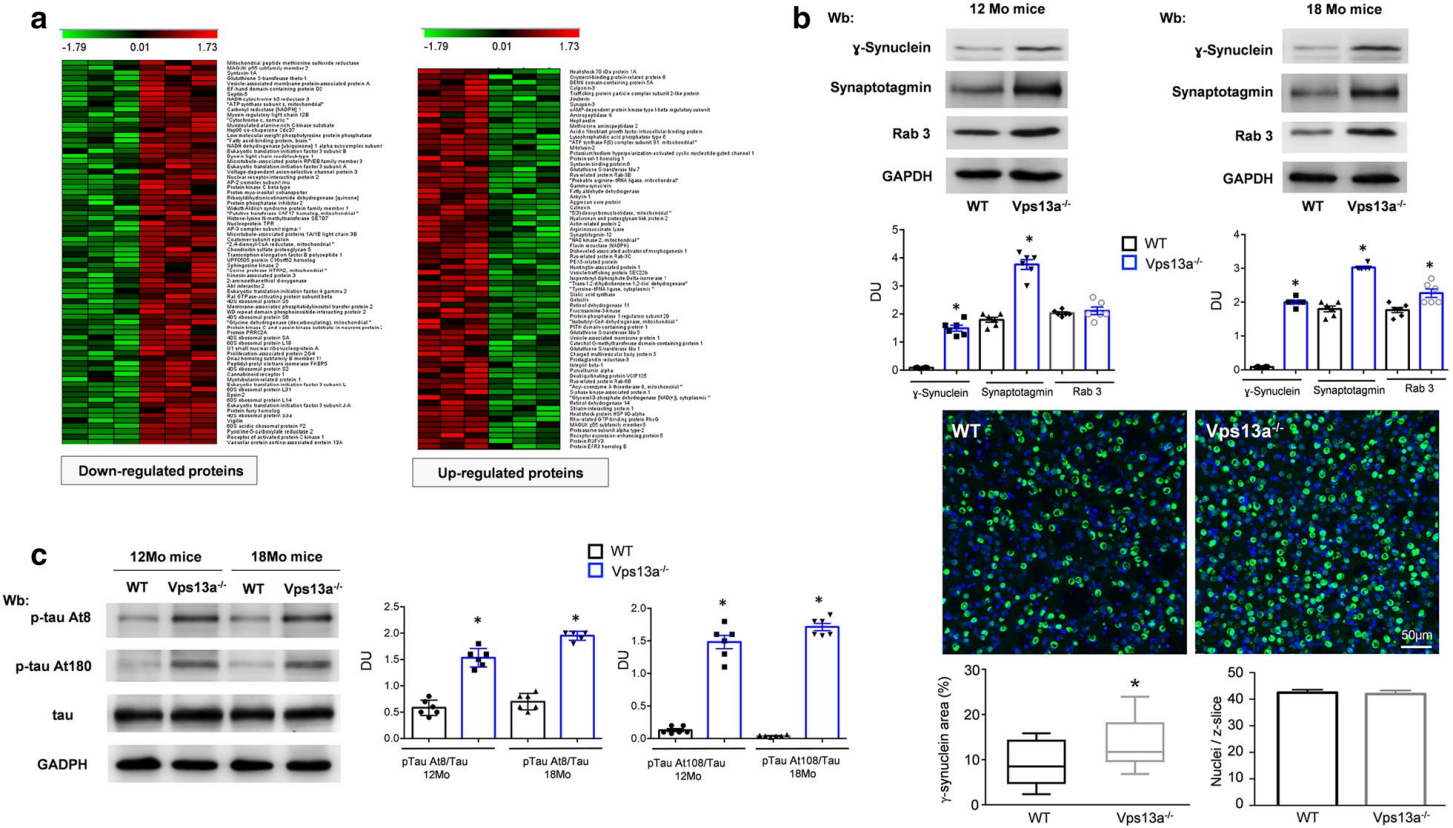
Proteomic analysis of Vps13a^−/−^ mouse basal ganglia revealed accumulation of neurotoxic proteins related to impaired autophagy. **a** Heatmaps of statistically relevant identified proteins. Each line corresponds to a protein and each column is relative to a different sample. The different coloration is dependent on quantity of protein present in sample based on the statistical performed analysis. Specifically, red color refers to up-regulated proteins while green is associated to down-regulated proteins. The logarithm Fold Change scale is also reported. Panel A and panel B report proteins down-regulated and up-regulated in Vps13a^−/−^ mice compared to WT, respectively. **b** Upper panel. Western blot (Wb) analysis of γ-Synuclein, synaptotagmin and Rab 3 in isolated basal ganglia from 12 and 18 months (Mo) old wild-type and Vps13a^−/−^ mice. GAPDH was the protein loading control. Middle panel. Densitometric analyses of the immunoblot bands similar to those shown are presented in bar graph. Data are means ± SEM (n = 6; * *P* < 0.02 ChAc vs. WT by t-test). Lower panel. Representative confocal images of the γ-synuclein protein (green) in the striatum of WT and Vps13a^−/−^ mice at 18 months of age. Boxplots summarize the results presented as mean ± standard deviation (*n* = 3 animals for group; * *P* = 0.029). **c** Western blot (Wb) analysis of phopho-tau At8, At180, and total tau in isolated basal ganglia from 12 and 18 months (Mo) old wild-type and Vps13a^−/−^ mice. GAPDH was the protein loading control. Densitometric analyses of the immunoblot bands similar to those shown are presented at right. Data are means ± SEM (n = 6; *p < 0.02 vs. WT by t-test)

To understand the abnormal proteostasis in the basal ganglia of Vps13a^−/−^ mice, we carried out proteomic analysis of Vps13a^−/−^ mouse basal ganglia, using a label-free differential proteomic analysis based on Spectral Counts quantification, using Rsc method [75]. Among 3351 total identified proteins, 143 were selected as statistically significant changeable of which 69 were downregulated and 74 were upregulated compared to the basal ganglia of wild-type animals (Fig. 4a). Among the up-regulated proteins, we found of interest the following proteins for our model: (1) γ-Synuclein, member of the synuclein family; and (2) Synaptotagmins, which are involved in regulation of synaptic vesicle exocytosis; and (3) Syntaxin-1, involved in synaptic transmission [6, 30, 47]. We confirmed the increased expression of γ-Synuclein and synaptotagmin in isolated basal ganglia from 12- and 18- months old Vps13a^−/−^ mice compared to wild-type animals (Fig. 4b). The accumulation of γ-synuclein was also confirmed by immunofluorescence microscopy (Fig. 4b, lower panel). The increased expression of γ-synuclein in basal ganglia from Vps13a^−/−^ mice is of specific note since mice genetically over-expressing γ-synuclein display an age-dependent neurodegeneration, abnormal psycho-emotional status and motor deficiency [47]. Increased levels of γ-synuclein have been reported in cerebrospinal fluid in Alzheimer disease (AD) patients, in individuals with Creutzfeld-Jakob disease [48], and may contribute to the pathogenesis of amyotrophic lateral sclerosis (ALS) [52]. We then asked whether the increase cellular levels of γ-synuclein might be associated with the accumulation of other neurotoxic proteins such as neuronal microtubule-associated protein tau, which organizes into pathogenic fibrils in phosphorylated form. We analyzed the Tau phospho-epitopes At8 and At180, which are reported to have functional importance in the neurodegeneration of Alzheimer’s disease [33]. In basal ganglia from Vps13a^−/−^ mice, we observed increased levels of At8 and At180 phosphorylated tau compared to wild-type animals (Fig. 4c). The accumulation of phosphorylated At8 and At180 tau protein has been previously linked with Lyn activity in the context of AD [18]: Remarkably, neither γ-synuclein nor tau phospho-epitopes At8 and 180 accumulated in isolated basal ganglia from Vps13a^−/−^ Lyn^−/−^ mice (Additional file 1: Fig. S7B-C). Taken together our evidence suggests ChAc as a novel disorder of proteostasis related to impaired autophagy with accumulation of neurotoxic proteins, supporting the rational to target active Lyn as a novel therapeutic option for ChAc.

### Dasatinib ameliorates hematologic but not neurologic disease markers in Vps13a^−/−^ mice

Since we tested dasatinib in our previous in vitro studies, we administrated dasatinib (50 mg/kg once a day for 4 weeks) to 12 months-old Vps13a^−/−^ mice. Dasatinib-treated Vps13a^−/−^ mice developed mild anemia associated with a significant reduction in reticulocyte count (Hb vehicle: 14.6 ± 0.8 vs. Hb dasatinib: 10.3 ± 1.5 g/dL; n = 8; *p* < 0.02; retic vehicle 390 ± 62 cell/µL vs. retic dasatinib: 201 ± 84 cell/µL n = 6; *p* < 0.05). In Vps13a^−/−^ mouse RBCs, dasatinib markedly reduced the amount of active Lyn and improved autophagy as supported by the reduction of Ulk1 accumulation compared to vehicle treated animals (Additional file 1: Fig. S8A, B). However, basal ganglia of dasatinib-treated Vps13a^−/−^ mice revealed no major difference in accumulation of active Lyn nor in levels of beclin-1 or beclin-1 related proteins such as Vps34 or Rab 5, suggesting a lacking effect of dasatinib in the central nervous system (Additional file 1: Fig. S8C, D). Mass spectrometric analyses with detection limit of 0.01 pg/µl detected no dasatinib in basal ganglia (Additional file 1: Fig. S9, Table S3). We therefore reviewed the literature on Lyn inhibitors that have been previously reported to better cross the BBB [12, 21, 28, 49, 67]. We focused on nilotinib, a second-generation TKI targeting Lyn, which has been previously shown to ameliorate mouse model phenotypes of PD and AD by improving autophagy [12, 21, 28], and has tested in 6 months phase II clinical trial in PD patients [49, 62].

### Nilotinib reduces acanthocytosis, improves autophagy and neuroinflammation in basal ganglia from Vps13a^−/−^ mice

**Fig. 5:**
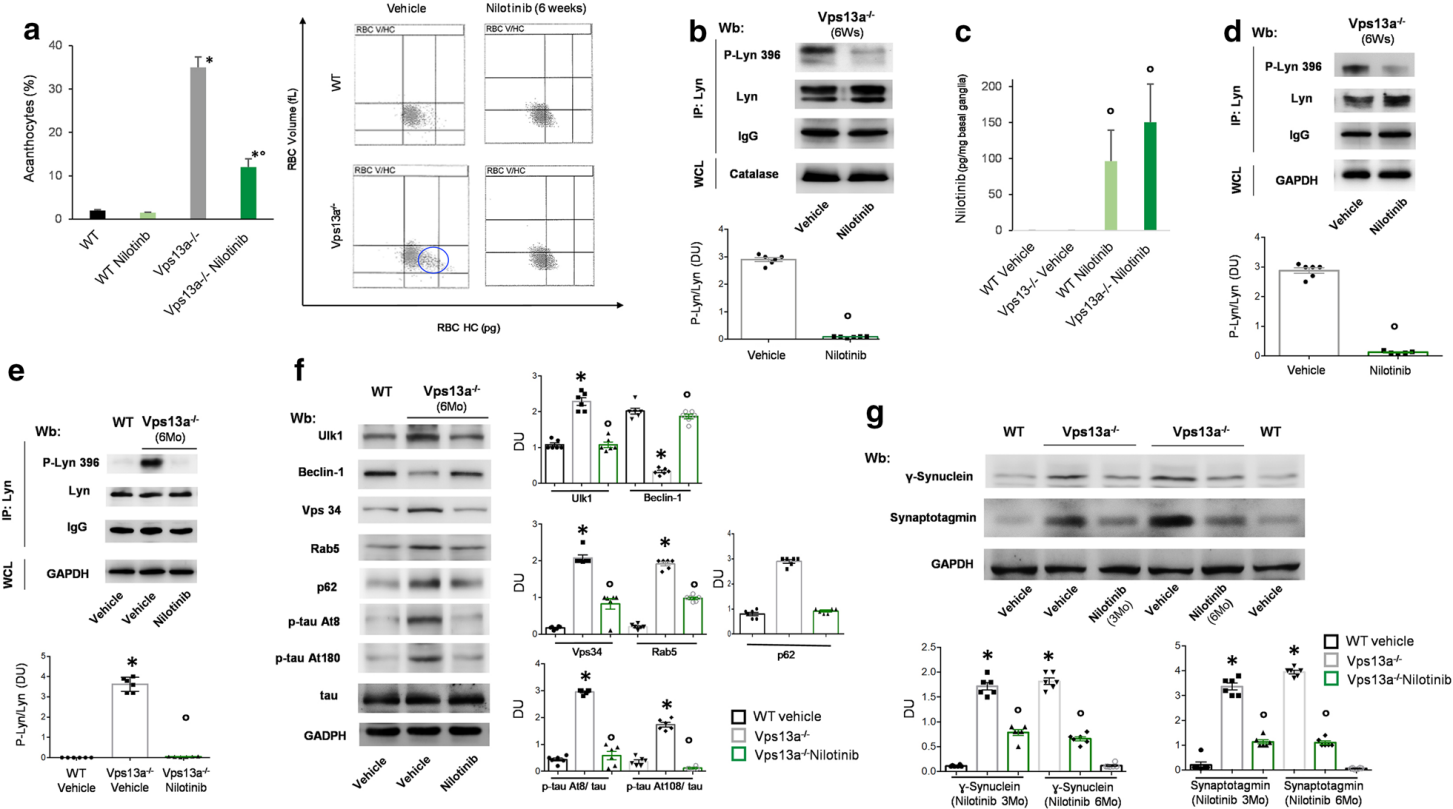
Nilotinib ameliorates Vps13a^−/−^ mouse red cell features and its passage into brain across the BBB prevents Lyn activation and improves autophagy. **a** Left panel. Quantitation of acanthocytes by brightfield microscopic analysis on Vps13a^−/−^ and Vps13a^−/−^ mice treated with nilotinib (25 mg/kg/d for 6 weeks). Data from 50 visual fields was collected by two blinded researchers. Results are means ± SEM n = 6; *P < 0.05 versus WT; °*P* < 0.05 versus vehicle treated Vps13a^−/−^ by 2-way ANOVA with Bonferroni correction for multiple comparison. Right panel. Red cell distribution histograms generated for red blood cell volume (RBC Volume) and cell hemoglobin concentration (RBC-HC) of RBCs from wild-type (WT) control, *Vps13a*^−/−^ mice treated with nilotinib (25 mg/kg/d for 6 weeks). One experiment representative of six others with similar result is shown. The blue circle indicates the presence of a subpopulation of dense red cells, containing acanthocytes as described in human patients (see also Lupo et al. [42]). **b** Total Lyn was immunoprecipitated from red cell cytosol fractions of Vps13a^−/−^ mice treated with vehicle or with nilotinib (25 mg/kg/d for 6 weeks (6Ws)) and detected with antibody against active Lyn (phospho-Lyn 396) or antibody against total Lyn (Wb: Western-blot). The experiment shown is representative of 6 experiments. IgG is used as loading control as catalase in whole cell lysate (WCL). Lower panel. Densitometric analysis of the immunoblots; means ± SEM (n = 6; *P* < 0.05 vs. WT by t-test). **c** Quantification of nilotinib in isolated basal ganglia from wild-type (WT) and *Vps13a*^*−/−*^ mice treated either with vehicle or nilotinib. Data are means ± SD (n = 6; °*P* < 0.05 vs. vehicle treated Vps13a^−/−^ by 2-way ANOVA for multiple comparison). **d** Total Lyn was immunoprecipitated from basal ganglia of Vps13a^−/−^ mice treated with vehicle or with nilotinib (25 mg/kg/d for 6 weeks (6Ws)). The experiment shown is representative of 6 experiments, each from an individual Vps13a^−/−^ mouse and each with similar results. IgG and catalase are used as loading control. WCL: whole cell lysate. Lower panel. Densitometric analysis of the immunoblots; means ± SEM (n = 6; °*P* < 0.05 vs. WT by t-test). **e** Total Lyn was immunoprecipitated from basal ganglia of wild-type and Vps13a^−/−^ mice treated with vehicle or with nilotinib (25 mg/kg/d for 6 months (6Mo), 12 months old mice) and detected with antibody against active Lyn (phospho-Lyn 396) or antibody against total Lyn (Wb: Western-blot). The experiment shown is representative of 6 experiments, each from an individual Vps13a^−/−^ mouse and each with similar results. IgG is shown as loading control as well as GAPDH in whole cell lysate (WCL). Lower panel. Densitometric analysis of the immunoblots; means ± SEM (n = 6; *P < 0.05 vs. WT; °*P* < 0.05 vs. vehicle treated Vps13a^−/−^ by 2-way ANOVA with Bonferroni correction for multiple comparison). **f** Western blot (Wb) analysis of Ulk1 (Atg1), Beclin-1, Vps34, Rab5, p62, phospho-tau At8, and At180 and total tau in isolated basal ganglia from 18 months old wild-type, and Vps13a^−/−^ mice treated with either vehicle or nilotinib (25 mg/kg/d for 6 months (6Mo)). GAPDH was used as loading control (See Additional file 1: Fig. 14S for data on nilotinib treated 12 months-old mice). Right panel. Densitometric analyses of the immunoblot bands similar to those shown are presented at right. Data are means ± SEM (n = 6; *P < 0.05 vs. WT; °*P* < 0.05 vs. vehicle treated Vps13a^−/−^ by 2-way ANOVA with Bonferroni correction for multiple comparison). **g** Western blot (Wb) analysis of γ-Synuclein and Synaptotagmin in isolated basal ganglia from 12 months old wild-type, and Vps13a^−/−^ mice treated with either vehicle or nilotinib (25 mg/kg/d for 3 months (3Mo)) and 18 months old wild-type, and Vps13a^−/−^ mice treated with either vehicle or nilotinib (25 mg/kg/d for 6 months (6Mo)). GAPDH was used as loading control. Lower panel. Densitometric analyses of the immunoblot bands similar to those shown are presented. Data are means ± SEM (n = 6; *P < 0.05 vs. WT; °*P* < 0.05 vs. vehicle treated Vps13a^−/−^ by 2-way ANOVA with Bonferroni correction for multiple comparison)

**Fig. 6:**
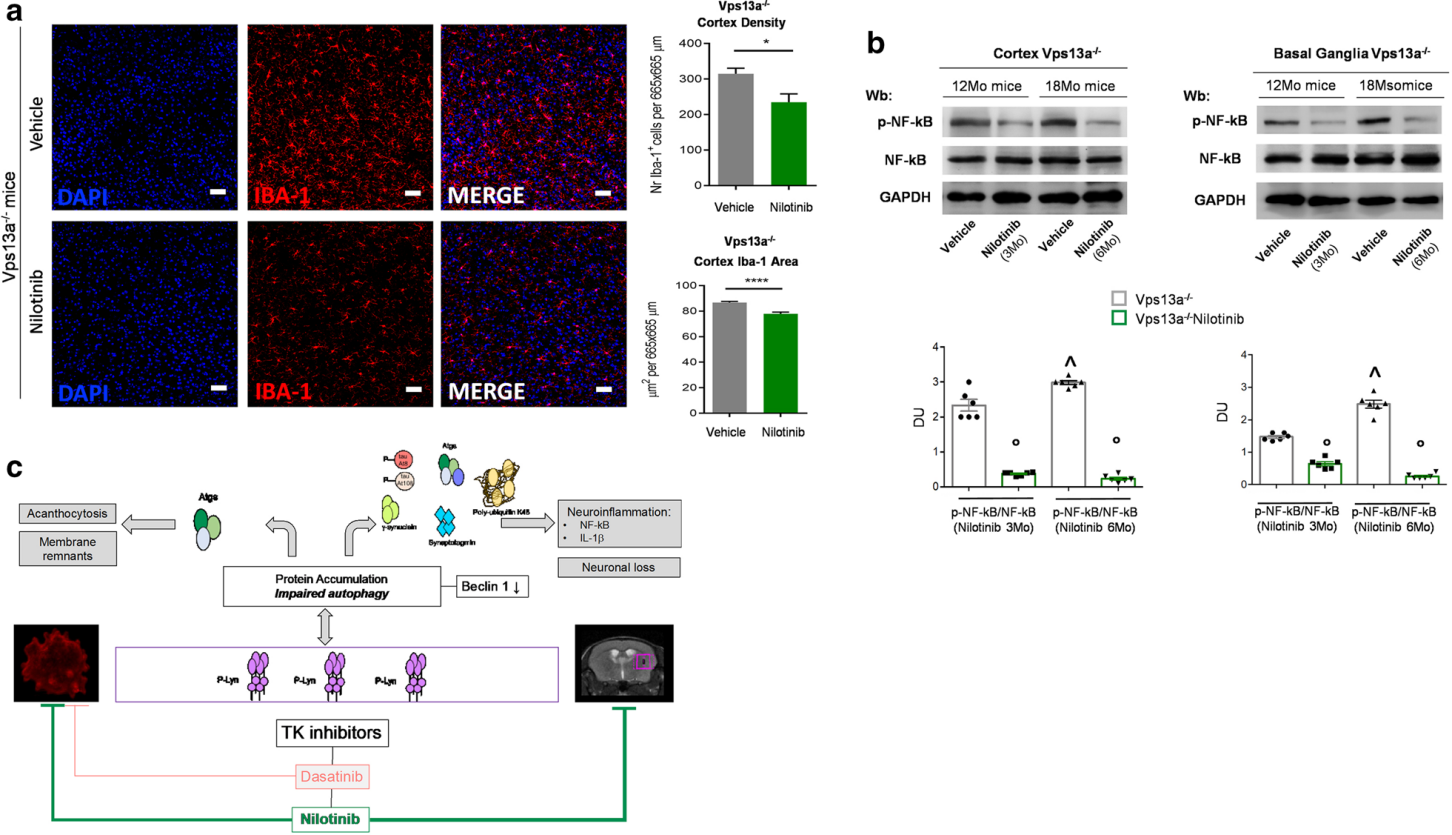
Nilotinib decreases neuroinflammation in Vps13a^−/−^ mice. **a** Representative images of Iba-1 positive microglia cells in cortex of Vps13a^−/−^ mice treated with vehicle or with nilotinib (25 mg/kg/d for 6 months) (Microglia in red, nuclei in blue). Scale bar:50 mm. Quantitative analyses show significant differences in microglial density and activation in the cortex of Vps13a^−/−^ vehicle compared to treated mice. Results are expressed as mean ± SEM (*P < 0.05; ****P < 0.0001; Unpaired t-test). **b** Western blot (Wb) analysis of phospho-NF-kB p65 and NF-kB p65 in the cortex (left panel) and in isolated basal ganglia (right panel) from 12 and 18 months (Mo) old wild-type mice and Vps13a^−/−^ animals treated with vehicle or with nilotinib (25 mg/kg/d for 3 months (3Mo) and 6 months (6Mo) respectively). GAPDH was the protein loading control. Lower panel. Densitometric analyses of the immunoblot bands similar to those shown are presented. Data are means ± SEM (n = 6; ^*P* < 0.05 vs. 12 months old mice; °*P* < 0.05 vs. vehicle treated Vps13a^−/−^ by 2-way ANOVA with Bonferroni correction for multiple comparison). **c** Mice genetically lacking chorein (Vps13a^−/−^) display phenotype similar to patients with chorea-acanthocytosis (ChAc). We show protein accumulation and impaired autophagy in both red cells and basal ganglia from Vps13a^−/−^ mice. This is associated with neuronal loss, neuroinflammation and generation of circulating acanthocytes. Tyrosine kinase inhibitors (TKI) targeting Lyn kinase have been tested in Vps13a^−/−^ mice. Nilotinib but not dasatinib reduces protein accumulation and ameliorates autophagy with reduction in neuronal loss and neuroinflammation as well as in circulating acanthocytes**.** Atgs: autophagy related proteins

In Vps13a^−/−^ mice, nilotinib (1) markedly reduced acanthocytosis and dense red cell fraction (Fig. 5a, Additional file 1: Fig. S10A); (2) prevented the accumulation of active Lyn in Vps13a^−/−^ mouse RBCs (Fig. 5b); and (3) normalized RBC autophagy related proteins Ulk1 and Atg7 accumulation (Additional file 1: Fig. S10B). Nilotinib was detected by LC–MS/MS spectrometric analysis in basal ganglia from WT and *Vps13a*^*−/−*^ mice (Fig. 5c, Additional file 1: Fig. S10C, Table S4). The presence of nilotinib in *Vps13a*^*−/−*^ mouse basal ganglia was associated with markedly reduced basal ganglia accumulation of active Lyn after nilotinib treatment for 6 weeks or for 6 months (Fig. 5d, e). Accumulation of the key autophagy-related proteins Ulk1, Vps34, Rab5 and p62 were also reduced in basal ganglia of mice treated with nilotinib for either 6 weeks or 6 months (Fig. 5f, Additional file 1: Fig. S10D). Noteworthy, increased *Vps13a*^*−/−*^ mouse basal ganglia beclin-1 levels were noted only after 6 months’ nilotinib treatment (Fig. 5f, Additional file 1: Fig. S10D). Accumulation of phospho-tau At8 and At180 (Fig. 5f, Additional file 1: Fig. S10E), γ-synuclein and synaptotagmin (Fig. 5g) were prevented by nilotinib treatment in basal ganglia from *Vps13a*^*−/−*^ mice. Finally, we evaluated the impact of long-term treatment (3–6 months) on neuroinflammation in Vps13a^−/−^ mice. As shown in Fig. 6a, b and Additional file 1: Fig. S11, we observed a significant reduction in microglia associated with decreased activated NF-kB p65 in both cortex and basal ganglia. Taken together, our data support the accumulation of active Lyn and impaired autophagy as possible therapeutic targets for clinical intervention in ChAc. We demonstrated that nilotinib crosses the BBB, improves autophagy and reduces neuroinflammation in a mouse model for ChAc.

## Discussion

ChAc is a rare, progressive, multisystem neurodegenerative disease of young adulthood with no available treatment to halt or retard its devastating progression out. The identification of new therapeutic option(s) targeting disease mechanism(s) represents an urgent unmet need in ChAc. We first characterized *Vps13a*^*−/−*^ mice in order to advance understanding of ChAc and to search for additional drug targets and novel drug candidates. Vps13a^−/−^ mice display (1) acanthocytes; (2) signs of both hyper- and hypokinetic movement disorders; (3) accumulation of active Lyn and of autophagy-related proteins in RBCs; and (4) RBC retention of remnants of double membrane and multivesicular bodies. Abnormalities in motor behavior observed in Vps13a^−/−^ mice correlate with the movement disorders, specifically dystonia, seen in ChAc patients [50, 70]. Indeed, the neurologic phenotype of *Vps13a*^*−/−*^ mice resembles that of another mouse model for ChAc carrying a deletion of exons 60–61. [66]. In isolated basal ganglia of Vps13a^−/−^ mice, we found signs of neurodegeneration associated with (1) accumulation of Lyn, stabilized in high molecular weight complexes; (2) accumulation of autophagy related proteins; and (3) reduction in expression of beclin-1, a key initiator of autophagy, due to increased caspase 3 activity. Normalization of phenotypes in the *Vps13a*^*−/−*^*Lyn*^*−/−*^ double knock out model substantiates the central role of accumulation of active Lyn in the pathophysiology of ChAc. Lyn has been previously reported to contribute to varied neuronal functions throughout the phosphorylation of key substrates such as NMDA or AMPA receptors [16, 41, 59]. In addition, in vitro studies also suggest that active Lyn might reduce or alter exocytosis by phosphorylation of both proteins of synaptic vesicles and actin cytoskeleton [16]. Lyn and Src family kinases have been also shown to participate in autophagy by targeting various autophagy-related proteins such as Ulk1 [36, 37, 42]. However, the accumulation of active Src family kinases might be per se cytotoxic, contributing to impaired autophagy as reported in cancer cells [72, 73] The accumulation of At8- and At180-phosphorylated tau proteins and γ-synuclein as well as of polyubiquinated proteins in Vps13a^−/−^ mouse basal ganglia is consistent with abnormal autophagy in the absence of chorein. Our data adds ChAc, for the first time, to the group of classical neurodegenerative proteinopathies such as AD, PD and HD. These three diseases are also characterized by abnormalities in autophagy as well as by reduced beclin-1 [58, 61, 63]. In addition, recent reports of severe neurodegeneration and impairment of autophagy in beclin1^−/−^ mice further links beclin-1-dependent autophagy to neurodegenerative diseases [45, 55, 65]. In particular, the beclin-1-Vps34 complex is critical for autophagosome formation, subsequently involving Atg14 with recruitment of Rab 5 [45, 58]. In Vps13a^−/−^ mice, the absence of chorein results in impairment of beclin-1 pathway with accumulation of Vps34, Atg14 and Rab5, suggesting a perturbation of protein trafficking associated with possible abnormalities in maturation and/or degradation of autophagosomes. This is further supported by the association between chorein with beclin-1 observed in basal ganglia from wild-type mice. Although chorein does not contain a recognized actin-binding motif, it carries a coil-coil binding motif that may be involved in beclin-1 interactions. A complex connection has been reported between beclin-1 and inflammation [24, 46]. In vitro studies show that block of autophagy results in up-regulation of pro-inflammatory cytokines such as IL-1β [24, 46]. Here, we found neuroinflammation in Vps13a^−/−^ mice associated with activation of NF-kB p65 and increased expression of IL-1β further emphasizing similarities between ChAc and other neurodegenerative disorders characterized by abnormal proteostasis such as PD or AD [15]. At this stage we cannot determine whether neuroinflammation is directly involved in the etiology of ChAc or indirectly related to the neurodegenerative processes in the basal ganglia. Although systematic investigation of ChAc neuropathology in human brains is still lacking, preliminary reports on brain from patients with ChAc suggests the presence of microgliosis [38, 39, 50].

Collectively these findings led us to test in our mouse ChAc model a Lyn kinase inhibitor that crosses the BBB more easily than dasatinib. Nilotinib beneficially impacts ChAc mouse hematological phenotype and improves ChAc RBC features. Furthermore, nilotinib was detected in basal ganglia from Vps13a^−/−^ mice resulting in improvement of autophagy with reduction of active Lyn and accumulation of autophagy related proteins. *Vps13a*^*−/−*^ mice treated long-term with nilotinib exhibited increased levels of basal ganglia beclin-1, associated with reduced microglia density and reduced active NF-kB p65. These data further supported the link between beclin-1 dependent autophagy and inflammation in ChAc mice.

In conclusion, our data show for the first time that the pathogenesis of ChAc is linked to perturbation of beclin-1 pathway, resulting in impaired autophagy with accumulation of active Lyn and classic neurotoxic proteins such as γ-synuclein or phospho-tau At8 and At180. Abnormal autophagy contributes to NF-kB activation and expression of pro-inflammatory cytokines, such as Il-1b, in combination with microglia activation, sustaining a neuroinflammatory environment in ChAc as in other neurodegenerative disorders such as PD or AD. Our data prove active Lyn to be a key regulator of neurodegeneration in ChAc, thus generating a rationale to consider TKIs targeting Lyn per se as possible and safe novel therapeutic approach for ChAc patients (Fig. 6c). Our results propose BBB-permeable Lyn kinase inhibitors such as nilotinib as first-line treatment options for patients suffering from ChAc. As nilotinib is already in clinical use for treatment of other diseases [21], larger-scale studies of nilotinib for treatment of ChAc should be eligible for accelerated approval. Our data support the proposal to repurpose nilotinib as new therapeutic option for ChAc.

## Supplementary Information

**Additional file 1.**
