# Supplementary material for: Therapeutic targeting of Lyn kinase to treat chorea-acanthocytosis
Source: Acta Neuropathol Commun. 2021 May 3;9:81. doi: 10.1186/s40478-021-01181-y (PMC8091687; doi:10.1186/s40478-021-01181-y)
Supplement: Supplementary file 1 — Additional file 1. [file 40478_2021_1181_MOESM1_ESM.docx]

**SUPPLEMENTARY MATERIALS**

**Supplementary Figures**

**
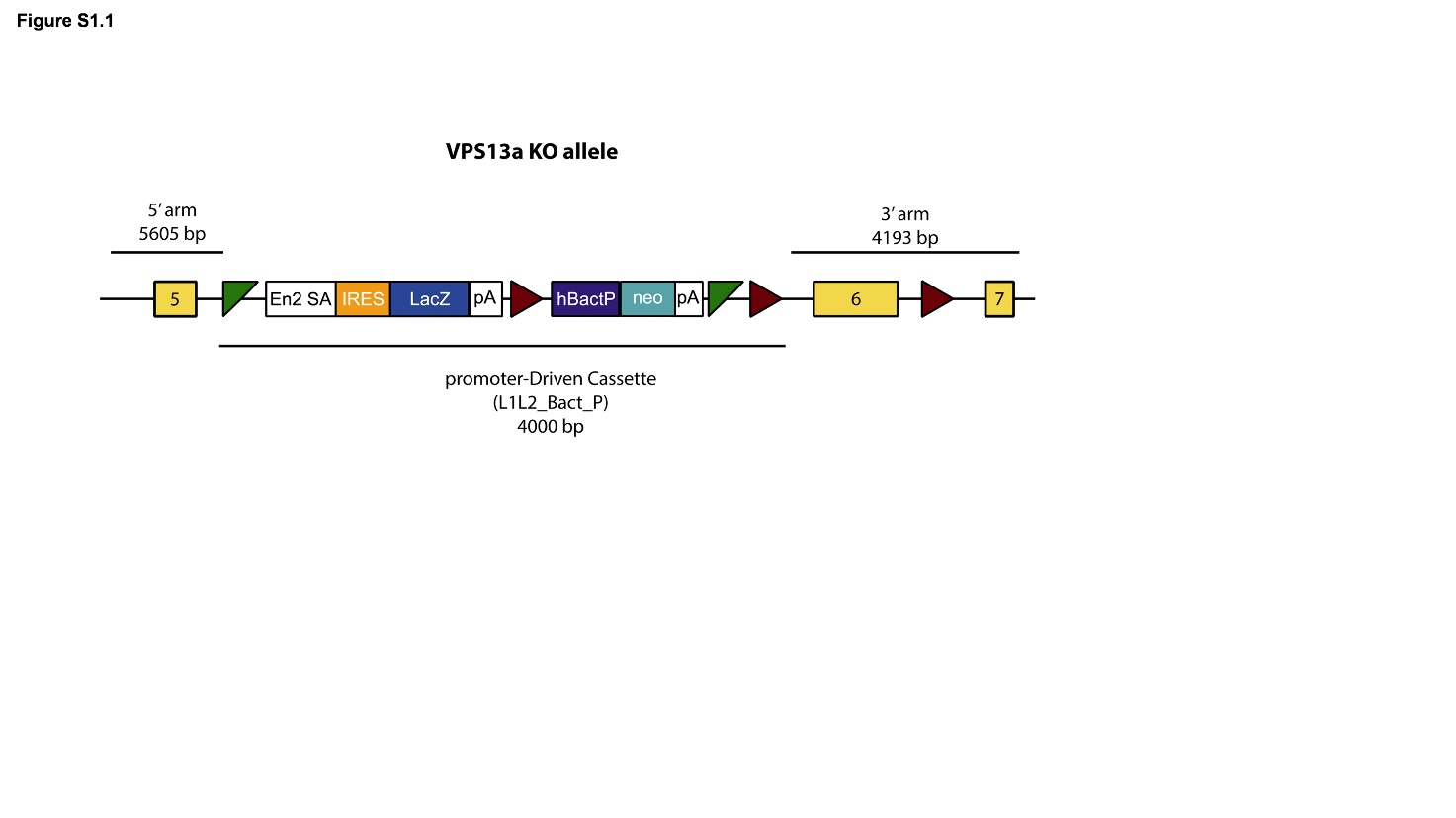
Figure S1.1 Schematic representation of the VPSA13A Knock Out allele.** The KO allele was obtained by insertion of the L1L2_Bact_P cassette at the position 16754850 (Build GRCm38) of Chromosome 19 upstream exon 6.

**
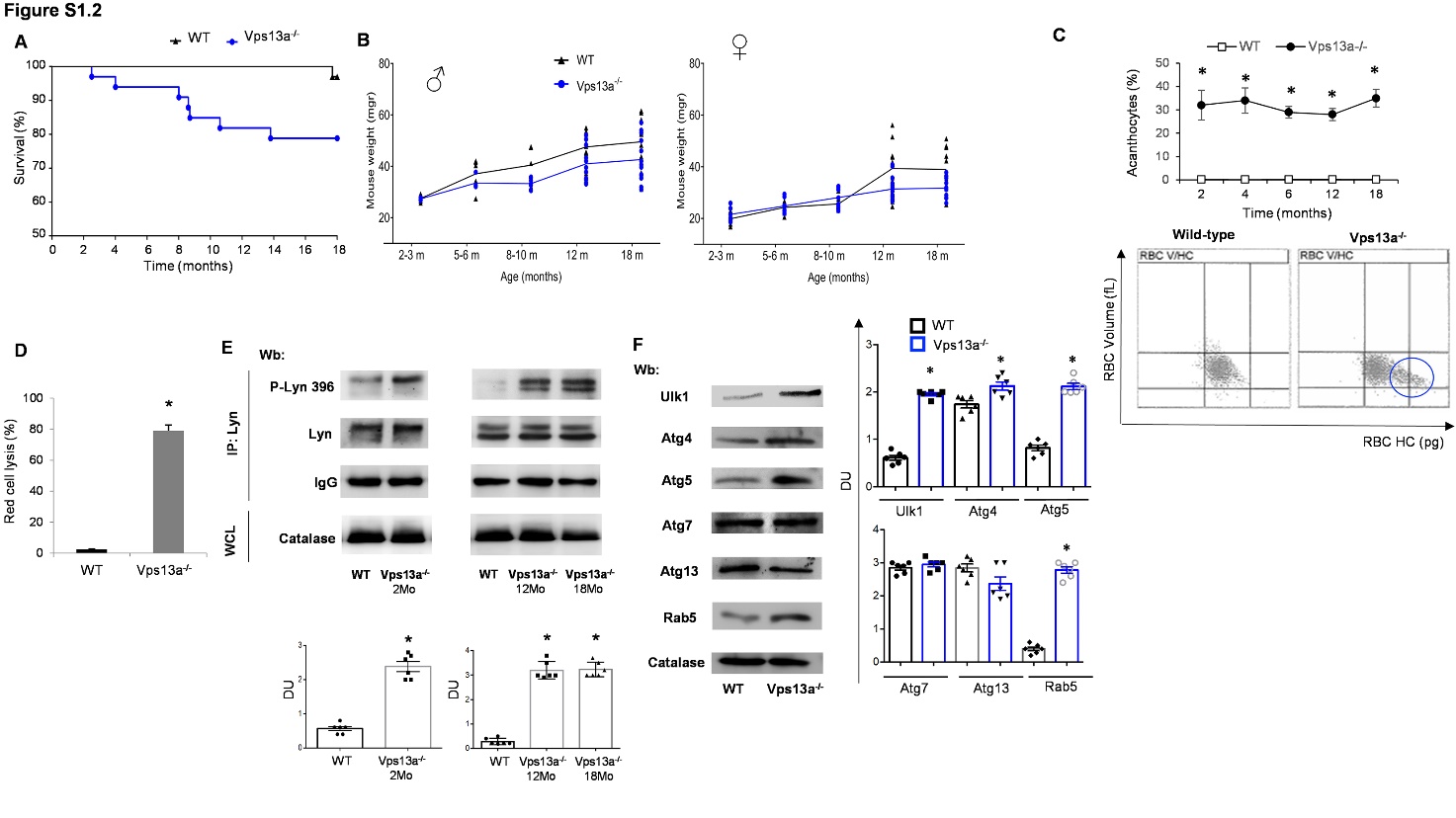
Figure S1.2 red cell features in *Vps13a^-/-^* mice. (A)** Survival of Vps13a^-/-^ and wild-type mice was assessed by long-rank test analysis (n=33 for each strains). **(B)** Weight during aging of male (♂) and female (♀) mice from wild-type (WT) and *Vps13a^-/-^* strains. Data are shown as single values, lanes connect means. **(C)** Quantitation of acanthocytes by brightfield microscopic analysis on WT and Vps13a^-/-^ mouse RBCs. Data from 50 visual fields was collected by two blinded researchers. Results are means ± SEM n=5; *p<0.002 *vs* WT. **P* < 0.02 compared with WT by 2-way ANOVA with Bonferroni correction for multiple comparison. **Lower panel.** Red cell distribution histograms generated for red blood cell volume (RBC Volume) and cell hemoglobin concentration (RBC-HC) of RBCs from wild-type and in Vps13a^-/-^ mice. The single experiment shown is representative of seven additional with similar results. Related values for hemoglobin distribution width (HDW) are shown in Table 2. The blue circle indicates the presence of a subpopulation of dense red cells, containing acanthocytes as described in human patients (Lupo F et al. Blood 2016). **(D)** Red cell lysis (%) at 156 mOsm in WT and *Vps13a^-/-^* mice at 6 months of age (n=6 in each group). Data are means ± SEM, * *P*< 0.001 vs. WT by t-test. **(E)** Total Lyn was immunoprecipitated from red cell cytosol fractions of WT and Vps13a^-/-^ mice and detected with antibody against active Lyn (phospho-Lyn 396) or antibody against total Lyn (Wb: Western-blot). The experiment shown is representative of 6 experiments, each from an individual Vps13a^-/-^ mouse and each with similar results. IgG is shown as loading control as well as catalase in whole cell lysate (WCL). **Lower panel.** Densitometric analysis of the immunoblots; means ± SEM (n=6; **P* < 0.05 by t-test vs WT). **(F)** Western blot (Wb) analysis of Ulk1 (Atg1), Atg4, 5, 7, 13 and Rab 5 in cytosolic fractions from red cells of wild-type (WT) and Vps13a^-/-^ mice. Catalase was used as protein loading control. Densitometric analyses of the immunoblot bands similar to those shown are presented at right. Data are means ± SEM (n=6; **P* < 0.02 by t-test vs. WT).

**
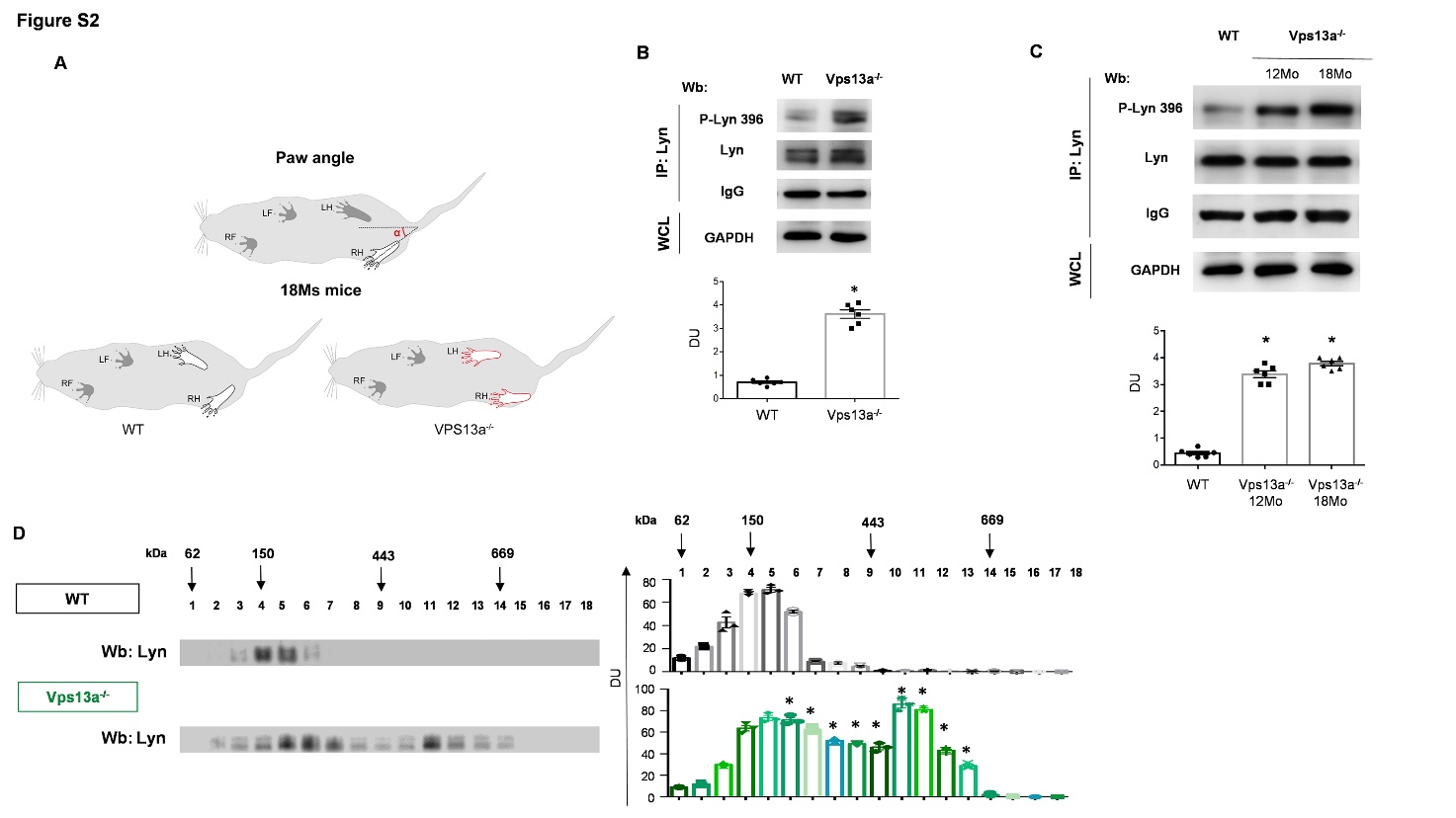
Figure S2. Activity of Lyn in basal ganglia from *Vps13a^-/-^* mice. (A) Top panel**. Outline of the parameter “paw angle” analysed with Catwalk® software. Paw angle is an estimate of the angle (α, in red) of the paw axis relative to the horizontal plane. **Lower panel**. Schematic representation of the deviation of the angle for both left and right paws (LH and RH, in red) in Vps13a^−/−^ mice compared to controls at 18 months of age. **(B)** Total Lyn was immunoprecipitated from detergent-solubilized isolated basal ganglia from 7 months old wild-type and *Vps13a^-/-^* mice and detected with antibody against active Lyn (phospho-Lyn 396) or total Lyn (Wb: Western blot). The representative experiment shown is one of 6 similar experiments with similar results. IgG was the loading control as well as GAPDH in whole cell lysate (WCL). **Lower panel**. Densitometric analysis of the immunoblots; data are means ± SEM (n=6; **P* <0.005 vs. WT by t-test). **(C)** Total Lyn was immunoprecipitated from basal ganglia of 18 months (Mo)-old wild-type (WT) and 12-18-months old *Vps13a^-/-^* mice and detected with antibody against active Lyn (phospho-Lyn 396) or antibody to total Lyn (Wb: Western-blot). The experiment shown is representative of 6 experiments, each from an individual *Vps13a^-/-^* mouse, with IgG as loading control. GAPDH was assayed in whole cell lysate (WCL). **Lower panel.** Densitometric analysis of the immunoblots; means ± SEM (n=6; P<0.05 vs. WT). **(D)** Cytosol from isolated basal ganglia from wild-type (WT) and Vps13a^-/-^ mice was loaded onto a linear glycerol gradient (10-40%) and centrifuged 18 hours at 100 000*g* in an SW60Ti rotor (Beckman Coulter) at 4°C. Eighteen fractions (200 μL each) were collected from each of the above gradients and analysed by immunoblotting with antibodies to Lyn. Arrows mark the glycerol gradient molecular mass standards: glutamate dehydrogenase (62 kDa), alcohol dehydrogenase (150 kDa), apoferritin (443 kDa), and thyroglobulin (669 kDa). The representative experiment shown is one of 6 similar experiments with similar results. Data are shown as means ± SEM (n=3); **P* < 0.05 compared with WT by 1-way ANOVA with Dunnett’s test for longitudinal comparison.

**
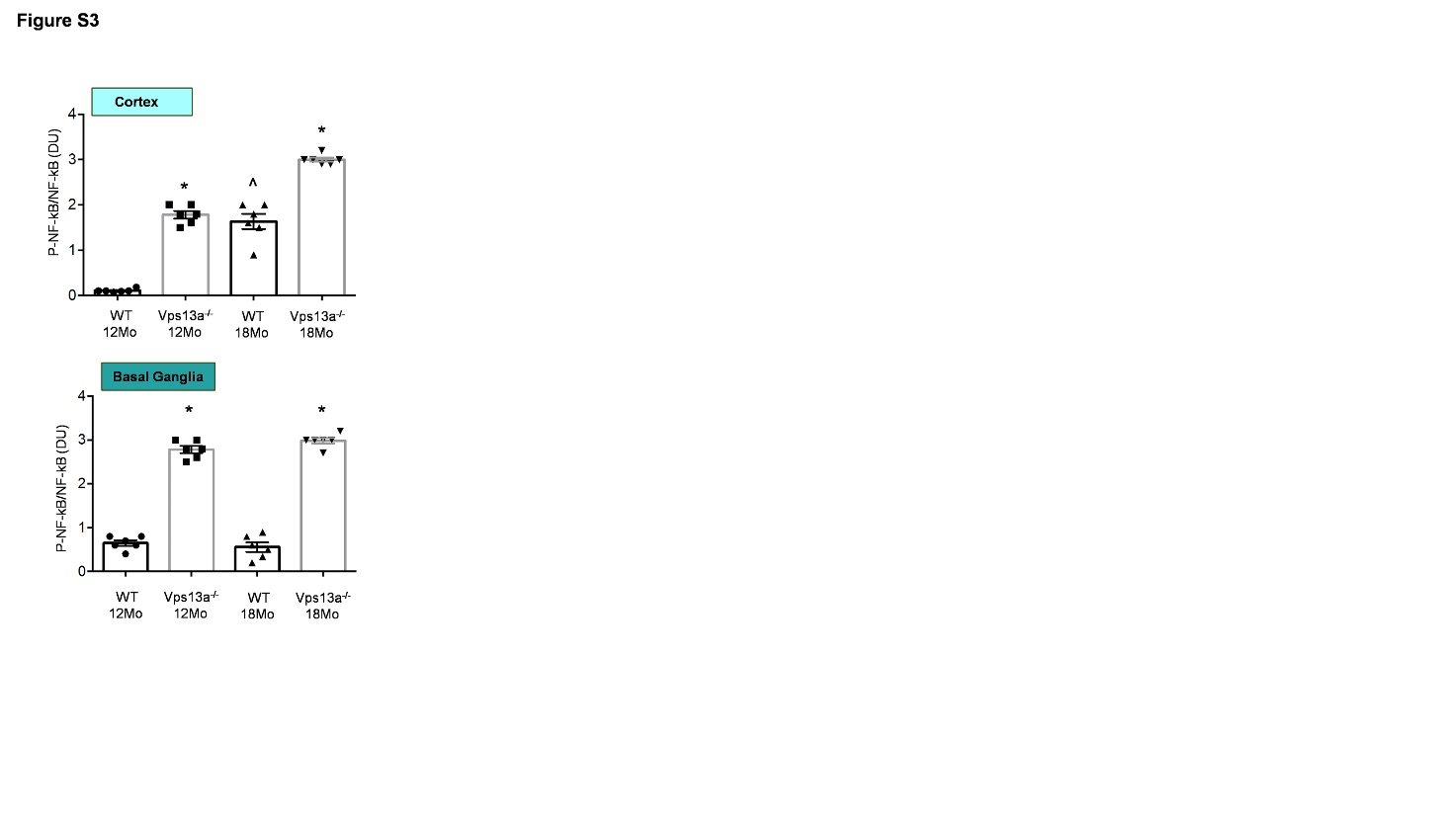
Figure S3. Activity of NF-kB in basal ganglia from *Vps13a^-/-^* mice.** Densitometric analyses of the immunoblot bands of Figure 3D. Data are means ± SEM (n=6; **P* <0.02 vs. WT; ^ *P*<0.05 12 months (Mo) old mice vs 18 months (Mo) old mice by two-way-ANOVA/Bonferroni’s multiple comparison test.

**
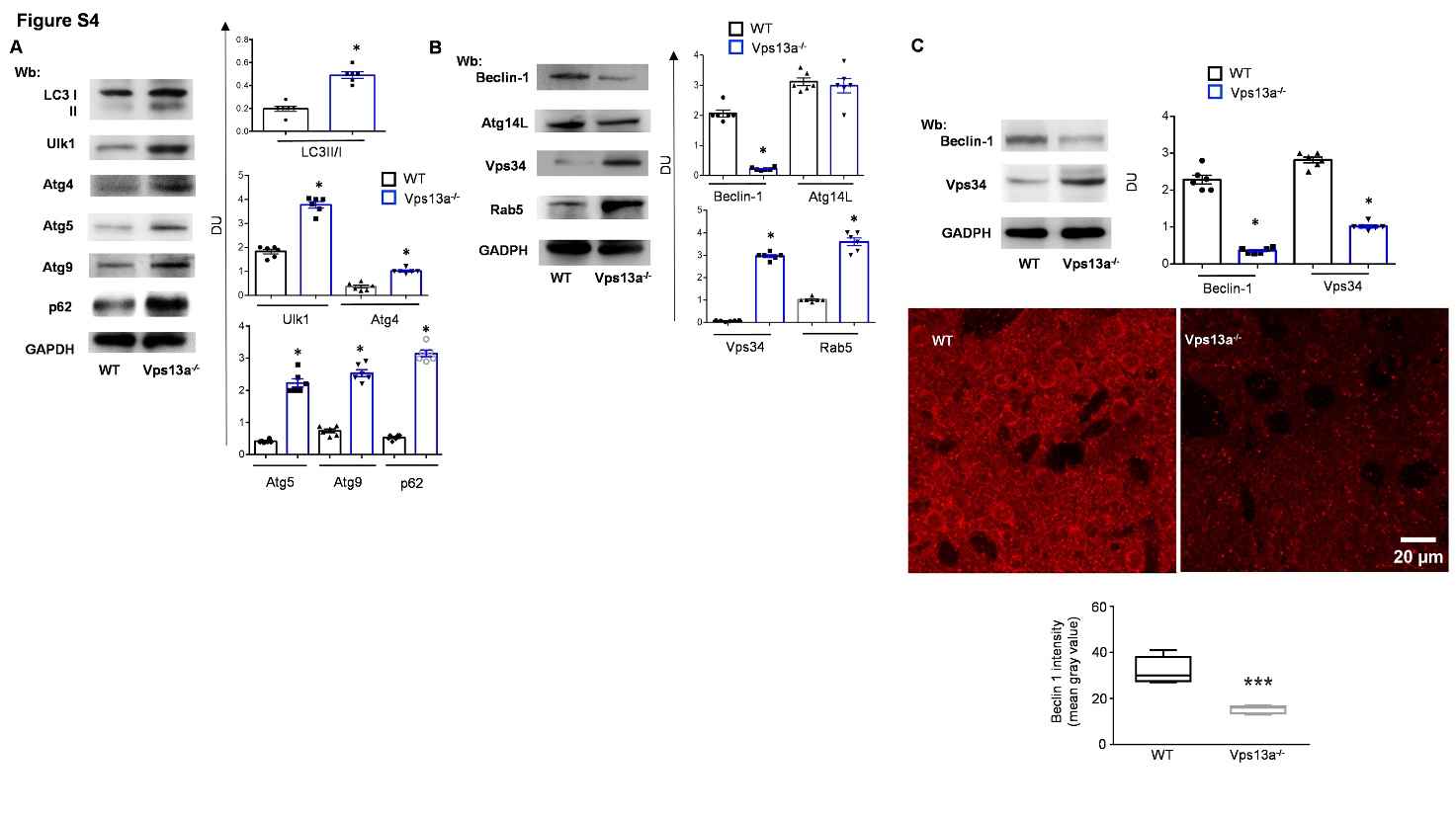
Figure S4. Impaired autophagy characterizes *Vps13a^-/-^ mouse basal ganglia.* (A)** Western blot (Wb) analysis of LC3I/II, Ulk1 (Atg1), Atg4, Atg5, Atg9 and p62 in isolated basal ganglia from 12-months (Mo)-old wild-type and *Vps13a^-/-^* mice. GAPDH was the protein loading control (See also Figure S8A for data on autophagy related proteins in 18 months old mice). Densitometric analyses of the immunoblot bands similar to those shown are presented at right. Data are means ±SEM (n=6; **P* <0.02 vs. WT by t-test). **(B)** Western blot (Wb) analysis of Beclin-1, Atg14L, Vps34, and Rab 5 in isolated basal ganglia from 12-months (Mo) old wild-type and Vps13a^-/-^ mice (See also Figure S8A for data on autophagy related proteins in 18 months old mice). GAPDH was the protein loading control. Densitometric analyses of the immunoblot bands similar to those shown are presented at right. Data are means ± SEM (n=6; **P* <0.02 vs. WT by t-test). **(C) Left panel.** Western blot (Wb) analysis of Beclin-1 and Vps34 in isolated basal ganglia from 18-month old wild-type (WT) and Vps13a^-/-^ mice. GAPDH was the protein loading control. **Right panel.** Densitometric analyses of the immunoblot bands similar to those shown are presented at right. Data are means ± SEM (n=6; **P* <0.02 vs. WT by t-test). **Lower panel.** Representative confocal images of the Beclin-1 protein (red) in the striatum of WT and Vps13a^-/-^ mice at 18 months of age. The box-plots shows the significantly lower intensity of Beclin-1 positive signal in transgenic animals. Data are means ± SEM (n=3 animals for group; ****P* <0.01 vs. WT).

**
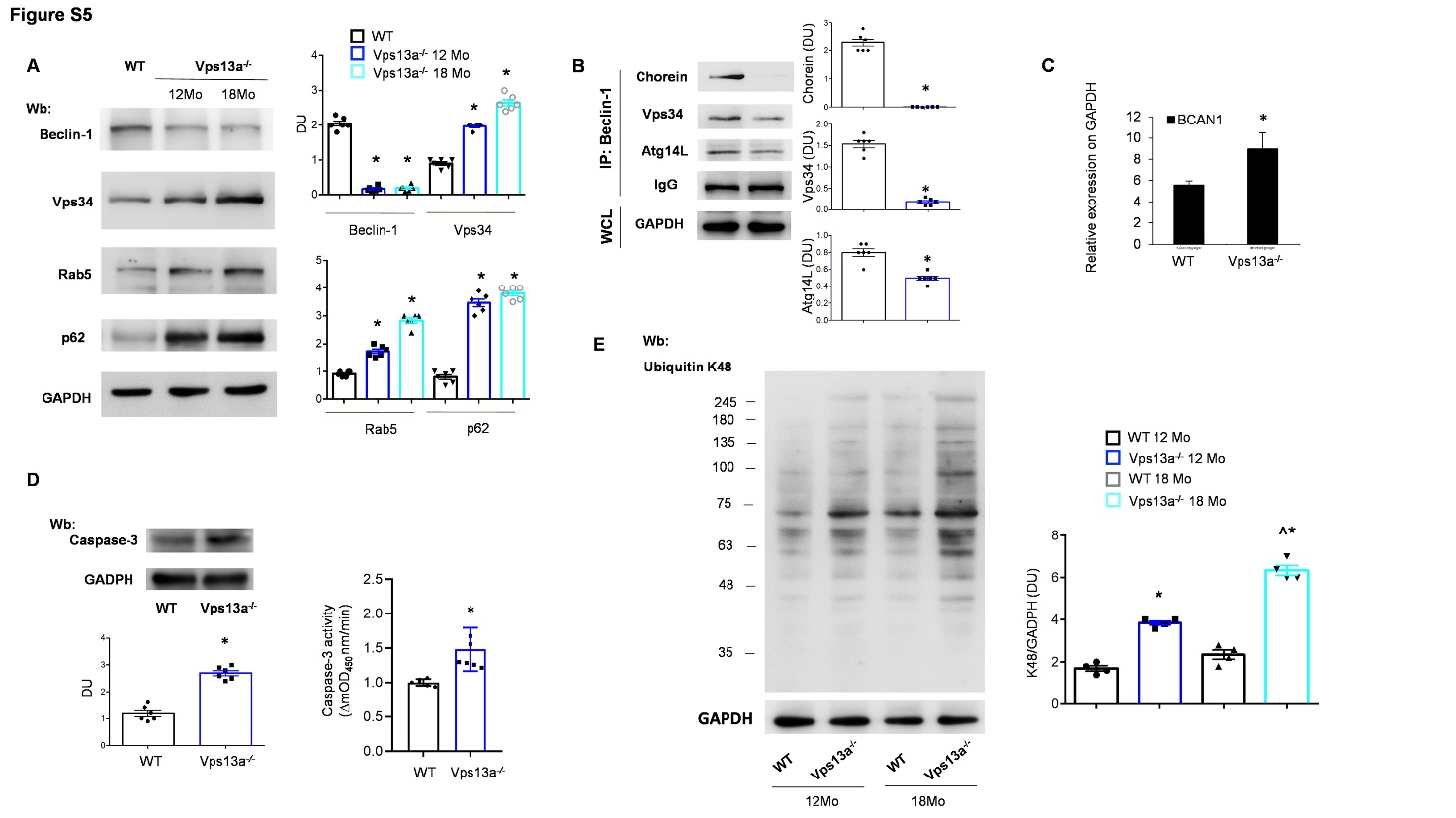
Figure S5. Impaired autophagy involves Beclin-1 pathway in *Vps13a^-/-^* mice.** **(A)** Western blot (Wb) analysis of Beclin-1, Vps34, Rab5, p62 in isolated basal ganglia of 18 months (Mo) old wild-type (WT) and 12-18-months (Mo) old *Vps13a^-/-^* mice. GAPDH served as protein loading control. Densitometric analyses of immunoblot bands like those shown are presented at right. Data are means ± SEM (n=6; **P*<0.02 vs. WT by two-way-ANOVA/Bonferroni’s multiple comparison test). **(B)** Beclin-1 was immunoprecipitated from isolated basal ganglia of 18-months-old wild-type and *Vps13a^-/-^* mice and detected with antibody against either chorein, Vps34 or Atg14L (Wb: Western blot). IgG was the protein loading control as well as GAPDH in whole cell lysate (WCL). Densitometric analyses of immunoblot bands like those shown are presented at right. Data are means ± SEM (n=6; * *P* <0.01 vs. WT by t-test). **(C)** Beclin-1 (BCAN1) expression as detected by RT-PCR in basal ganglia from 12-month old wild-type and *Vps13a^-/-^* mice. Data are shown as means ± SEM (n=6); *p<0.02 *Vps13a^-/-^* vs. WT by t-test. **(D)** **Left panel.** Western blot (Wb) analysis of caspase-3 in isolated basal ganglia from 12-month old wild-type and Vps13a^-/-^ mice. GAPDH was the protein loading control. Densitometric analyses of the immunoblot bands similar to those shown are presented at the bottom. Data are means ± SEM (n=6; **P*<0.05 ChAc vs, WT by t-test). **Right panel.** Caspase-3 activity in isolated basal ganglia from 12-months old wild-type and Vps13a^-/-^ mice. Caspase-3 activity was determined by the Caspase-3 Fluorometric Assay kit (according to the manufactures) (see also ref. Kang SJ et al J neuroscience 2003& Kaushal V et al 2014). Data are shown as means ± SEM (*n*=7); **P* <0.05 ChAc vs WT by t-test. **(E)** **Left panel.** Western blot (Wb) analysis of ubiquitinated proteins (K48) in basal ganglia isolated from 12-18-months (Mo) old wild-type (WT) and *Vps13a^-/-^* mice. GAPDH served as protein loading control. **Right panel.** Densitometric analyses of the immunoblot bands similar to those shown. Data are means ± SEM (n=6; * *P* <0.02 vs. WT; ^ *P* <0.05 compared to 12-months (Mo) old *Vps13a^-/-^* mice by two-way-ANOVA/Bonferroni’s multiple comparison test).

**
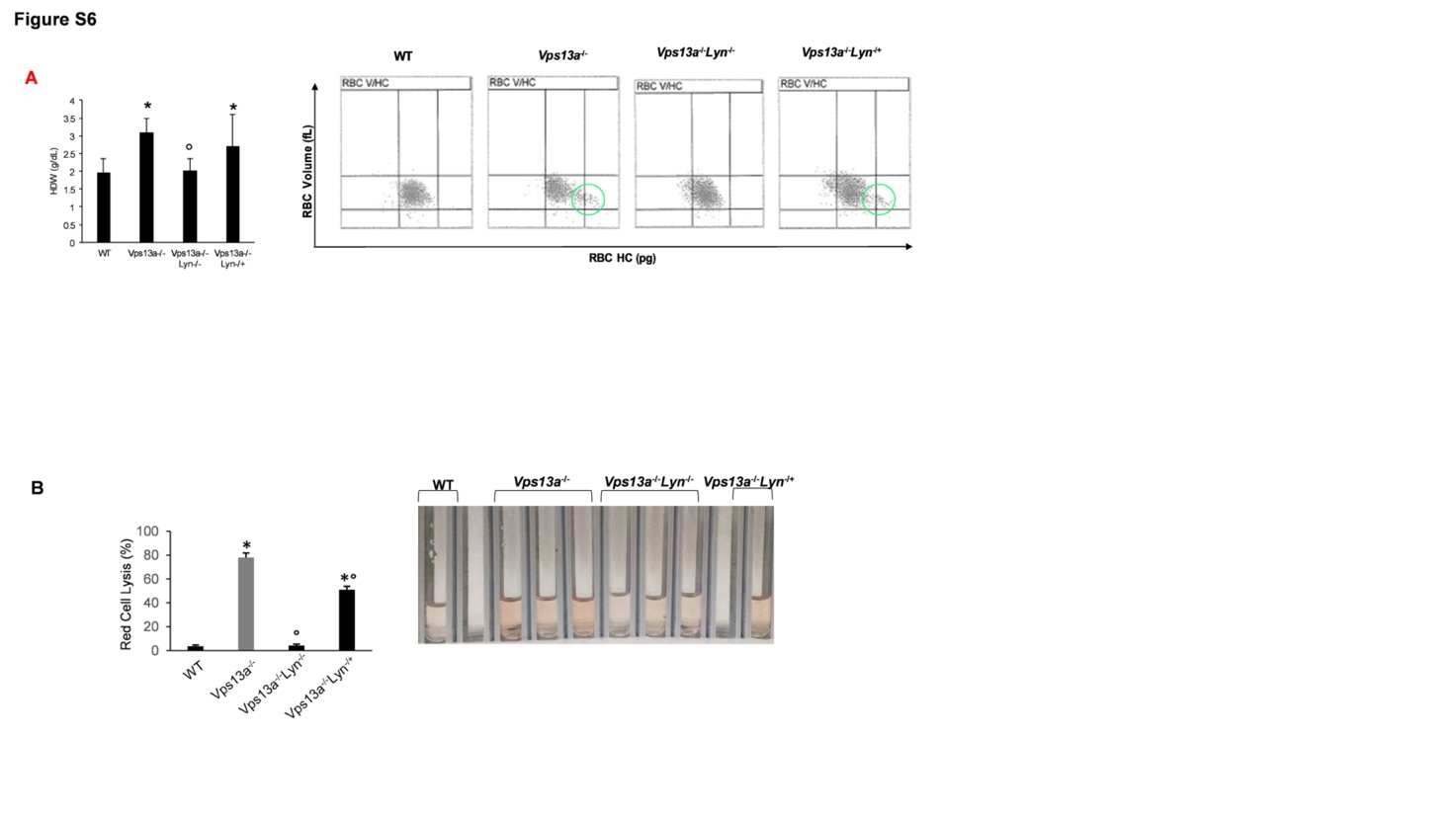
**

**Figure S6. Hematologic features of *Vps13a^-/-^Lyn^-/-^* mice. (A) Left panel.** Values for haemoglobin distribution width (HDW) are shown in bar graph (see Figure 3). Data are presented as means ± SEM (n=6 each strain; *P*< 0.001 vs WT. °*P*<0.002 vs *Vps13a^-/-^* by two-way-ANOVA/Bonferroni’s multiple comparison test). **Right panel**. Red cell distribution histograms generated for red blood cell volume (RBC Volume) and cell haemoglobin concentration (RBC-HC) of RBCs from wild-type, Vps13a^-/-^ , Vps13a^-/-^ Lyn^-/-^ and Vps13a^-/-^ Lyn^-/+^ mice. The single experiment shown is representative of six additional with similar results. The green circle indicates the presence of a subpopulation of dense red cells, containing acanthocytes. **(C)** Percentage of red cell lysis (%) at 156 mOsm in wild-type (WT) control, *Vps13a^-/-^* , *Vps13a^-/-^Lyn^-/-^* and *Vps13a^-/-^Lyn^-/+^* mice at 6 months of age (n=6 in each group). Data are means ± SEM * *P*< 0.001 vs WT. °*P* <0.002 vs *Vps13a^-/^* by two-way-ANOVA/Bonferroni’s multiple comparison test. **Lower panel.** One representative pictures of red cells lysis at 156 mOsm in wild-type (WT) control, *Vps13a^-/-^*, *Vps13a^-/-^Lyn^-/-^* and *Vps13a^-/-^Lyn^-/+^*.

**Figure S7. *Vps13a^-/-^Lyn^-/-^* mice display reduced accumulation of γ-synuclein and phospho- tau At8 and At108 proteins. (A)** Western blot (Wb) analysis of total Lyn, chorein in isolated basal ganglia of *Vps13a^-/-^* and *Vps13a^-/-^Lyn^-/-^* mice. GAPDH was used as protein loading control. Densitometric analyses of the immunoblot bands similar to those shown are presented at right. Data are means ± SEM (n=6; ° *P* <0.02 compared to *Vps13a^-/-^* mice by two-way-ANOVA/Bonferroni’s multiple comparison test). **(B)** Western blot (Wb) analysis of γ-synuclein in isolated basal ganglia of wild-type (WT), *Vps13a^-/-^* and *Vps13a^-/-^Lyn^-/-^* mice. GAPDH was used as protein loading control. Densitometric analyses of the immunoblot bands similar to those shown are presented at right. Data are means ± SEM (n=6; * *P* <0.02 vs. WT; ° *P* <0.02 compared to *Vps13a^-/-^* mice by two-way-ANOVA/Bonferroni’s multiple comparison test). **(B)** Western blot (Wb) analysis of phospho-tau At8 and At180 and total tau proteins in isolated basal ganglia of wild-type (WT), *Vps13a^-/-^* and *Vps13a^-/^Lyn^-/-^* mice. GAPDH was protein loading control. Densitometric analyses of the immunoblot bands similar to those shown are presented at right. Data are means ± SEM (n=6; **P*<0.02 vs. WT; ° *P* <0.02 compared to *Vps13a^-/-^* mice by two-way-ANOVA/Bonferroni’s multiple comparison test).

**
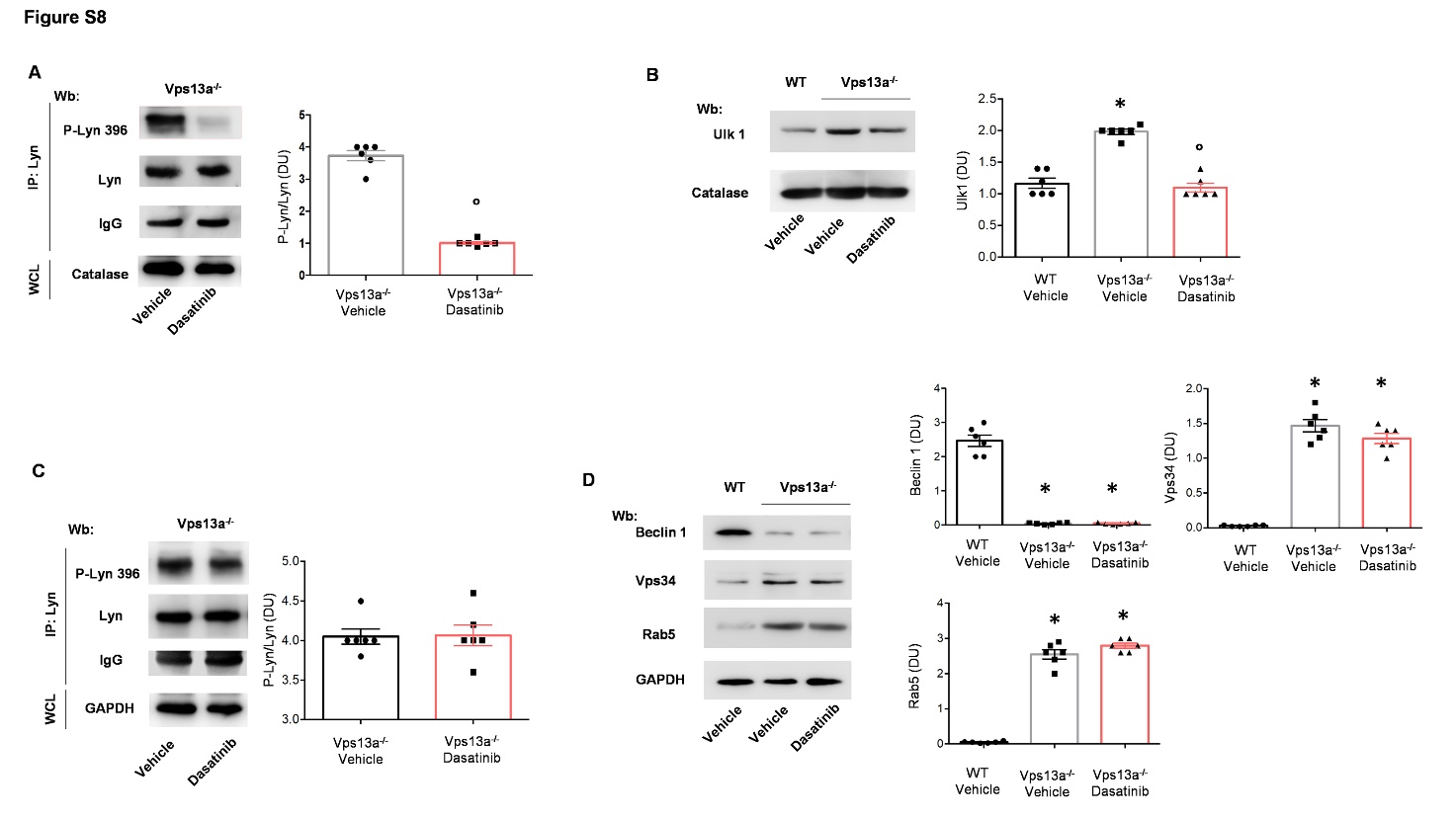
Figure S8.** **Effects of dasatinib on *Vps13a^-/-^* basal ganglia.** **(A)** Total Lyn was immunoprecipitated from red cells of *Vps13a^-/-^* mice treated with either vehicle or dasatinib and detected with antibody against active Lyn (phospho-Lyn 396) or antibody against total Lyn (Wb: Western blot). The experiment shown is representative of 4 experiments, each from individual *Vps13a^-/-^* mice and with similar results. IgG is loading control, as is Catalase in whole cell lysate (WCL). **Right panel.** Densitometric analysis of the immunoblots; means ± SEM (n=6; *P*<0.05 vs. WT by t-test). **(B)** Western blot (Wb) analysis of Ulk1 in red cell cytosol fraction from *Vps13a^-/-^* mice treated with either vehicle or dasatinib. Catalase was protein loading control. Densitometric analyses of the immunoblot bands similar to those shown are presented at right. Data are means ± SEM (n=6; *p<0.02 vs. WT; °p<0.02 compared to *Vps13a^-/-^* mice by two-way-ANOVA/Bonferroni’s multiple comparison test). **(C)** **Left panel.** Total Lyn was immunoprecipitated from isolated Vps13a^-/-^ basal ganglia of mice treated with either vehicle or dasatinib and detected with antibody against active Lyn (phospho-Lyn 396) or antibody against total Lyn (Wb: Western-blot). The experiment shown is representative of 6 experiments, each from an individual *Vps13a^-/-^* mouse and with similar results. IgG is loading control, as is catalase on whole cell lysate (WCL). **Right panel.** Densitometric analysis of the immunoblots; means ± SEM (n=6; P<0.05 vs. WT by t-test). **(D)** Western blot (Wb) analysis of Beclin-1, Vps34 and Rab5 in isolated basal ganglia from wild-type (WT) or *Vps13a^-/-^* mice treated with either vehicle or dasatinib. GAPDH served as protein loading control. Densitometric analyses of the immunoblot bands similar to those shown are presented at right. Data are means ± SEM (n=6; *p<0.02 vs. WT; °p<0.02 compared to *Vps13a^-/-^* mice by two-way-ANOVA/Bonferroni’s multiple comparison test).

**
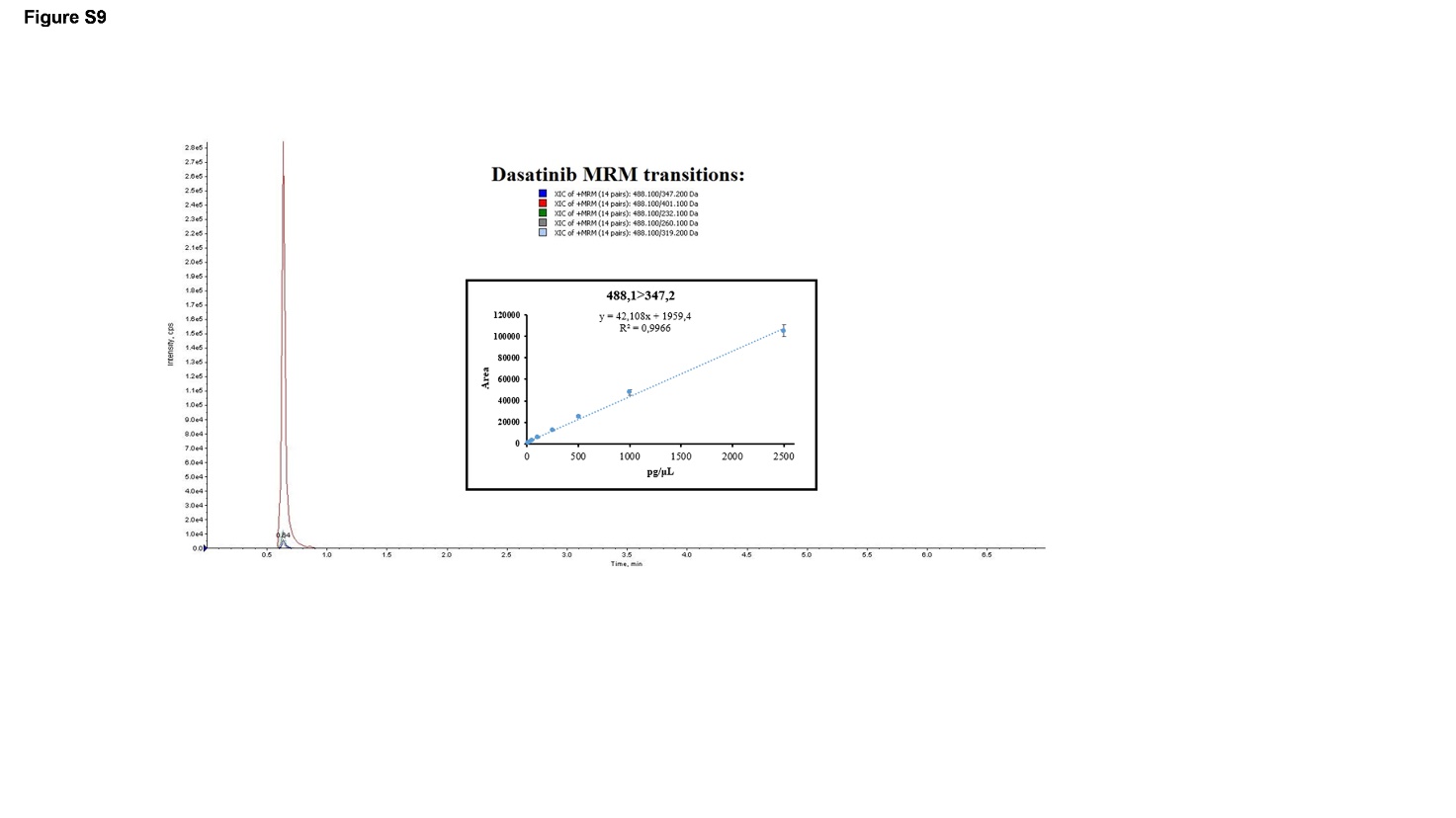
Figure S9. Representative MRM Chromatogram for Dasatinib and calibration curve**. Dasatinib was coeluted at 0.64 min and the calibration curve for m/z 488.1 → 347.2 quantifier transition was used to plot the values of the basal ganglia samples. The calibration curve was obtained in 10-2500 pg/μL concentration range.

**
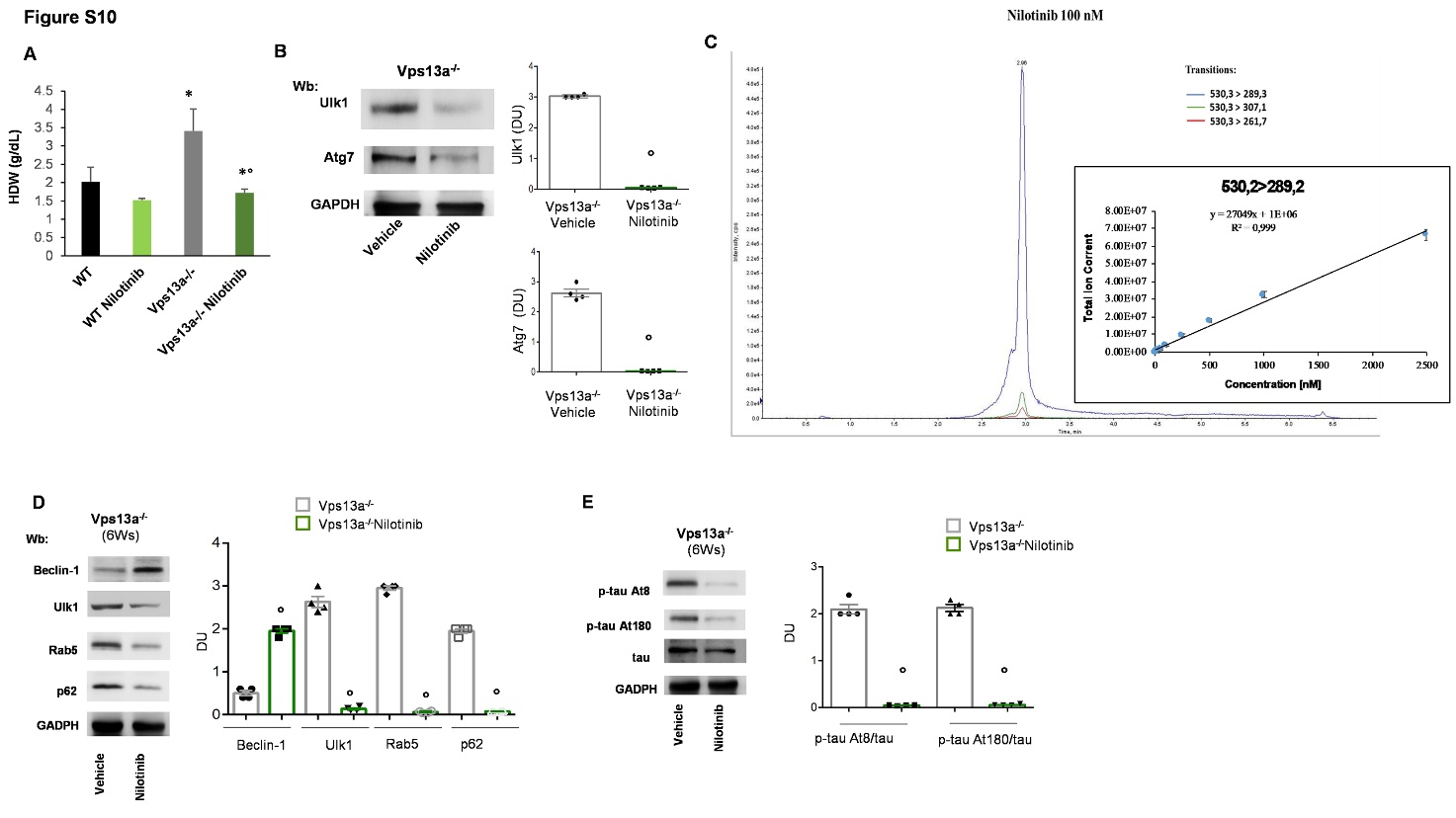
Figure S10. Effects of nilotinib on *Vps13a^-/-^* mice (A)** Haemoglobin distribution width (HDW) of RBCs from wild-type (WT) control or *Vps13a*^-/-^ mice treated with nilotinib (25 mg/kg/d for 6 weeks, 6 Ws). Data are presented as means ± SEM (n=6 each strain, *P<0.05 vs WT; °P<0.05 vs vehicle treated *Vps13a^-/-^* mice by two-way-ANOVA/Bonferroni’s multiple comparison test). **(B)** Western blot (Wb) analysis of Ulk1 (Atg1) and Atg7 in red cell cytosolic fraction from *Vps13a^-/-^* mice treated with either vehicle or nilotinib. GAPDH served as protein loading control. Densitometric analyses of the immunoblot bands similar to those shown are presented at right. Data are means ± SEM (n=4; °p<0.05 vs *Vps13a^-/-^* vehicle-treated mice by t-test). **(C)** Representative MRM Chromatogram for Nilotinib and calibration curve. For Nilotinib analysis the 530,2->289.2 transition was selected as quantifier. By plotting the area under the peak of the quantifier transition as a function of the concentration of the standards, it was possible to calculate the calibration curve for Nilotinib. The calibration curve was obtained in the 0.5-2500 nM concentration range. **(D)** Western blot (Wb) analysis of beclin-1, Ulk1 (Atg1), Rab 5 and p62 in basal ganglia from *Vps13a^-/-^* mice treated with either vehicle or nilotinib. GAPDH served as protein loading control. Densitometric analyses of the immunoblot bands similar to those shown are presented at right. Data are means ± SEM (n=4; °p<0.05 vs *Vps13a^-/-^* vehicle-treated mice by t-test). **(E)** Western blot (Wb) analysis of phospho-tau At8 and At108 and total tau proteins in basal ganglia from *Vps13a^-/-^* mice treated with either vehicle or nilotinib. GAPDH served as protein loading control. Densitometric analyses of the immunoblot bands similar to those shown are presented at right. Data are means ± SEM (n=4; °p<0.05 vs *Vps13a^-/-^* vehicle-treated mice by t-test).

**
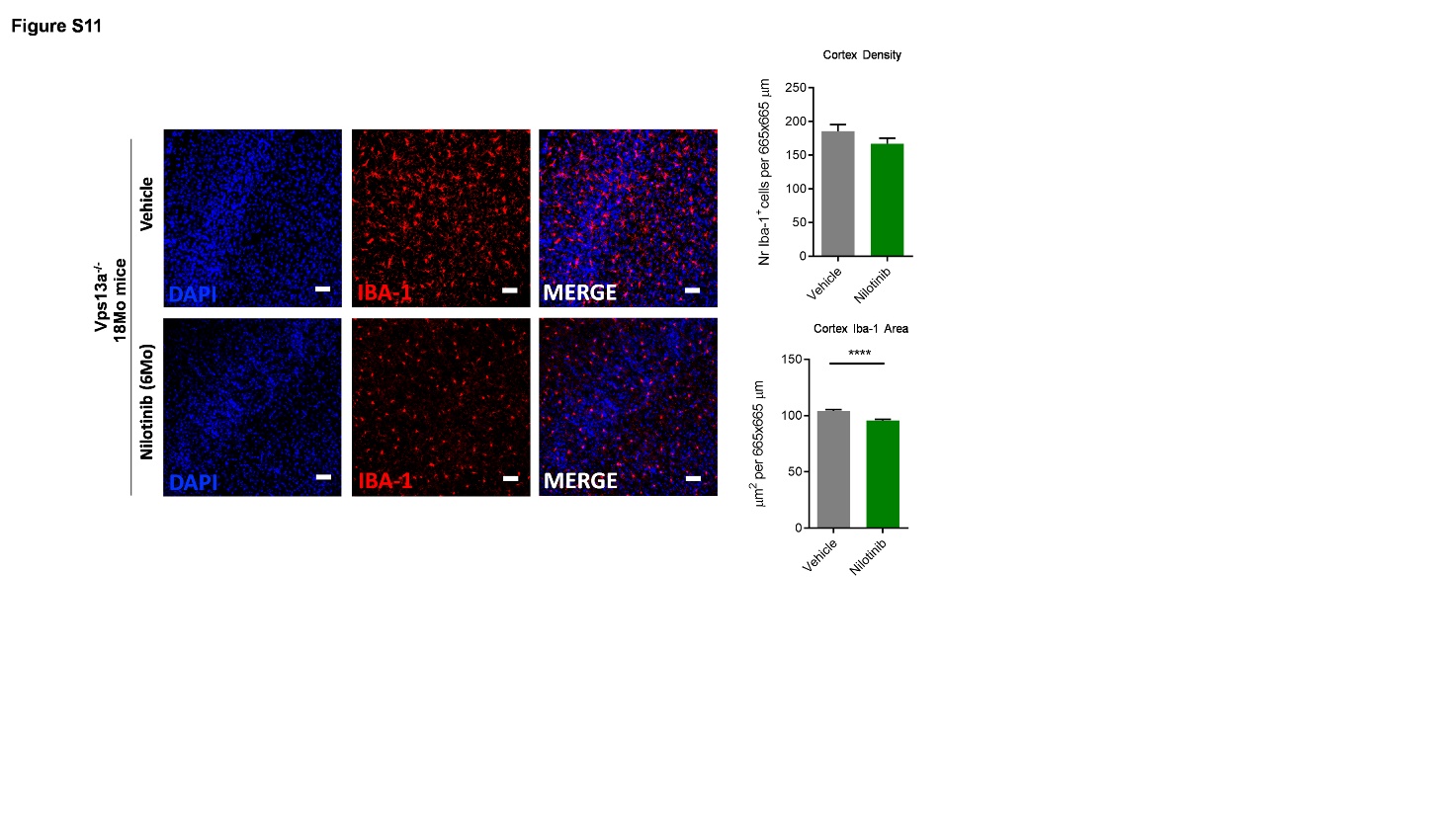
Figure S11. Nilotinib reduces microglia in *Vps13a^-/-^* mice.** Representative images of Iba-1 positive microglia cells in cortex of *Vps13a^-/-^* mice treated with vehicle or nilotinib (25 mg/kg/d for 6 months, 6Mo. Microglia in red. Nuclei in blue). Scale bar: 50mm. Quantitative analysis of microglia show significant differences in microglial activation in the cortex of *Vps13a^-/-^* mice treated with vehicle or nilotinib. Results are expressed as the mean ±SEM (****P<0.0001; Unpaired t-test).

**Supplementary tables**

| Table S1. Primers used in analysis of basal ganglia from *Vps13a^-/-^* and wild-type mice | | |
| --- | --- | --- |
| Gene | Forward Primer Sequence  (5’→ 3’) | Reverse Primer Sequence  (5’→ 3’) |
| Il-1β | GCTGAAAGCTCTCCACCTCAA | TTGTCGTTGCTTGGTTCTCCT |
| Bcan1 | ACCAATGTCTTCAATGCCACC | CAAGCGACCCAGTCTGAAAT |
| GAPDH | CCACATCGCTCAGACACCAT | AGTTAAAAGCAGCCCTGGTGAC |

| Table 2S. Hematological parameters and red cell indices in wild-type and Lyn^-/-^ mice | | |
| --- | --- | --- |
|  | **Wild-type mice**  **(*n*=3)** | **Lyn^-/-^ mice**  **(*n*=3)** |
| Hct (%) | 46.3 ± 1.2 | 44.4 $\pm$ 1.6 |
| Hb (g/dl) | 15.1 ± 0.7 | 14.1 $\pm$ 0.6 |
| Retics (%) | 3.4± 0.9 | 2.8 $\pm$ 0.4 |
| Hct: hematocrit; Hb: hemoglobin; Retics: reticulocytes | | |

| Table S3.  Compound-dependent parameters of dasatinib in HPLC-MRM/MS analysis | | | | | | |
| --- | --- | --- | --- | --- | --- | --- |
| Analyte | **Precursor ion (m/z)** | **Product ions (m/z)** | **Dwell time (ms)** | **DP (V)** | **CE (V)** | **CXP (V)** |
| Dasatinib | 488.1 | 401.1 | 100 | 78 | 25 | 30 |
|  |  | 347.2 |  |  |  |  |
|  |  | 319.0 |  |  |  |  |
|  |  | 260.1 |  |  |  |  |
|  |  | 232.1 |  |  |  |  |

| Table S4.  Compound-dependent parameters of nilotinib in HPLC-MRM/MS analysis | | | | | | |
| --- | --- | --- | --- | --- | --- | --- |
| Analyte | **Precursor ion (m/z)** | **Product ions (m/z)** | **Dwell time (ms)** | **DP (V)** | **CE (V)** | **CXP (V)** |
| Nilotinib | 530.3 | 289.2 | 100 | 84 | 39 | 30 |
|  |  | 261.3 |  |  |  |  |
|  |  | 307.1 |  |  |  |  |

**Supplementary materials and methods**

**VPS13A knock out mice.** The VPS13A knock out (KO) heterozygous mice in the C57BL/6N background were obtained from the EMMA consortium (<https://www.infrafrontier.eu/search?keyword=VPS13A&category=strains>; EMMA ID; EM:07284). The KO allele was obtained by insertion of the L1L2_Bact_P cassette at the position 16754850 (Build GRCm38) of Chromosome 19 upstream exon 6. The cassette is composed of an FRT site followed by the lacZ sequence and a loxP site. This first loxP site is followed by a neomycin resistance gene under the control of the human beta-actin promoter, SV40 polyA, a second FRT site and a second loxP site. Insertion of this cassette creates a reporter knockout mouse.

The homozygous mice were obtained by crossing two heterozygous mice. The pups were genotyped by PCR, using the following primers: for the WT

Vps13a_82078_F: ACCCTCCTCAGCATCTTCTATGTC;

Vps13a_82078_R: CAGAAAAGCCAACCATACAATCG, with expected size band of 541 base pair (bp); for the KO allele

Vps13a_82078_F ACCCTCCTCAGCATCTTCTATGTC

CAS_R1_Term TCGTGGTATCGTTATGCGCC, with expected size band of 386 bp.

**Immunofluorescence microscopy of ChAc red blood cells.**  Erythrocytes were stained with anti-ß-Actin-FITC-conjugated antibody (1:50; biorbyt) and Phalloidin-eFluor660 (1:100, eBioscience) to detect filamentous actin (F-actin) as described previously [8,6]. Confocal microscopy was performed and analysed with a Zeiss LSM 5 EXCITER confocal laser-scanning module (Carl Zeiss) and instrument software.

**Osmotic fragility assay.** Red cell osmotic fragility is increased in ChAc patients compared to healthy controls [21,3,4,12]. We were therefore able to evaluate osmotic fragility in EDTA blood using a single osmotic point of 158 mOsm. RBCs from healthy controls were always analyzed in parallel with ChAc red cells. We also evaluated osmotic fragility in mouse blood samples using a single osmotic point of 177 mOsm.

**Electron microscopy of erythrocytes.** Aliquots of RBC suspensions were gently pelleted by centrifugation and fixed for 30 min at 4° C in 2.0% glutaraldehyde (Electron Microscopy Science, FT. Washington, PA, USA) in 0.1M phosphate buffer. Cells were than washed three times in buffer and post-fixed with 1% osmium tetroxide and potassium ferricyanide (Merck, Darmstadt, Germany) for 45 min. All fixation procedures were at room temperature. Fixed pellets were dehydrated in graded acetones and embedded in a mixture of Epon and Araldite (Electron Microscopy Sciences, PA, USA). Ultrathin sections (silver interference colour) were cut with an Ultracut E ultramicrotome (Reichert-Jung, Vienna, Austria), placed on copper grids, stained with lead citrate, and imaged and photographed in a Philips Morgagni 268D electron microscope operated at 80 kV.

**Tissues molecular analysis.**

**Immunoblot analysis.** Frozen basal ganglia and cortex tissues from each studied group were homogenized and lysed in iced lysis buffer (HLB) containing 150 mM NaCl, 25 mM bicine, 0.1% SDS, 2% Triton X-100, 1 mM EDTA, protease inhibitor cocktail tablets (Roche), 1 mM Na_3_VO_4_ final concentration) then centrifuged 30 min at 4°C at 12,000 *g [18,9,5]*. Packed red cells were lysed in ice-cold phosphate lysis buffer (LB; 5 mM Na_2_HPO_4_, pH 8.0, containing protease inhibitor cocktail tablets, 3 mM benzamidine final concentration, 1 mM Na_3_VO_4_ final concentration) and centrifuged 10 min at 4 °C at 12,000 *g*. Red cell membrane (ghost) and cytosol fractions were obtained as previously reported [17,13,16]. Proteins were quantified and analysed by one-dimensional SDS–polyacrylamide gel electrophoresis. Gels were transferred to nitrocellulose membranes for immunoblot analysis with specific antibodies: anti-chorein VPS13A (HPA021662, Merck-Sigma, Darmstadt, Germany), anti-Catalase-Peroxisome marker (AbCam, Cambridge, UK), anti-Lyn (2732, Cell Signalling, USA), anti-phospho-Lyn (Y396) (p-Lyn 396; Cell Signalling, USA), anti-LC3B (NB600-1384, Novus Biological Europe, Abingdon, UK), anti-Ulk1 (H-240, Santa Cruz Biotechnology, Inc, USA), anti-APG7 (Atg7, 3617, ProSci Inc., USA), anti-Atg13 (5799, ProSci Inc., USA), anti-Atg 4b (I-13, Santa Cruz Biotechnology, Inc, USA), anti-Atg5 (D5F5U, Cell Signaling Technology, Leiden, NL), Atg9A (AbCam, Cambridge, UK), anti-Atg14L (SAB3500814, Merck-Sigma, Darmstadt, Germany), anti-Rab5 (AbCam, Cambridge, UK), anti-Rab3A (AbCam, Cambridge, UK), anti-NFkB-phospho-S536 (93H1) (Cell Signaling Technology, Leiden, NL); anti-NFkB p65 (C22B4) (Cell Signaling Technology, Leiden, NL), anti-p62 (AbCam, Cambridge, UK), anti-beclin1 (AbCam, Cambridge, UK ), anti-Vps34 (NB110-87320, ProSci Inc., USA), anti-pro-Caspase 3 (AbCam, Cambridge, UK ), anti-ɣ-Synuclein (AbCam, Cambridge, UK ), anti-synaptotagmin (ASV30) (AbCam, Cambridge, UK), anti-p-Tau Ser202-Thr205 (AT8, Thermo Fisher Scientific, USA), anti p-Tau Thr231 (AT180, Thermo Fisher Scientific, USA), anti-Tau (HT7, Thermo Fisher Scientific, USA), anti-Ubiquitin (linkage-specific K48) (AbCam, Cambridge, UK), and anti-GAPDH (D-6, Santa Cruz Biotechnology, Inc, USA). Secondary donkey anti-rabbit IgG and anti-mouse IgG HRP conjugated were from GE Healthcare Life Sciences (Little Chalfont, UK). Secondary donkey anti-goat IgG HRP conjugated was from SCBT. Secondary donkey anti-rat IgG HRP conjugated was from AbCam. Blots were developed with Luminata Forte Chemiluminescent HRP Substrate from Millipore (Billerica, MA, USA), and images were acquired with the Image Quant Las Mini 4000 Digital Imaging System (GE Healthcare Life Sciences). Densitometric analyses used ImageQuant TL software (GE Healthcare Life Sciences).

**Immunoprecipitation assay.** Immunoprecipitation (IP) assays were carried out as previously reported [12,4], using anti-Lyn antibody (Cell Signalling, USA) and anti-Beclin1 antibody (AbCam, Cambridge, UK).

**Quantitative RT-PCR.** Protocols used for RNA isolation, cDNA preparation, and quantitative RT-PCR have been previously described [14,15]. Detailed primer sequences are available on request and shown in Supplementary Table 1S**.**

**Behavioural tests.**

**Anxiety trait assessment.**Anxiety trait was assessed in mice at 12 month-old mice using the elevated-plus maze (EPM). In the EPM test, the animal’s spontaneous tendency to explore the environment is contrasted by the natural preference towards more confined, protected locations. Mice were placed at the center of a cross-shaped polycarbonate maze for 5 minutes. For each subject, the number of entrances and the time spent in the center or in either the open or the closed arms are recorded. An arm is considered to have been entered when the center-point of the rodent was in that arm. Video-recordings were scored with the aid of the Ethovision XT^®^ (Noldus^®^, Wageningen, The Netherlands) software. The amount of time spent in the closed arms relative to the open arms is regarded as a measure of anxiety trait.

**Spontaneous locomotor activity assessment**. Spontaneous locomotor activity was assessed using a PhenoTyper^®^ system (Noldus^®^, Wageningen, The Netherlands) when mice were 12 months old. Mice were individually housed in the PhenoTyper cages (30 x 30 x 35 cm) equipped with an infrared camera on the top, under a standard 12:12 h light/dark cycle, with controlled temperature (~22 - 24 °C) and *ad libitum* food and water. After one week of adaptation to the PhenoTyper cage, mice were continuously video recorded for two days in undisturbed conditions. Video-recordings were analysed offline. The locomotor activity of mice was tacked with the aid of the fully automated tracking Ethovision XT^®^ (Noldus^®^, Wageningen, The Netherlands) software.

**Gait and motor performance assessment**. Quantitative assessment of gait and motor performance were performed in both strains of mice at 12 and 18 months of age by the CatWalk^TM^ system (Noldus^®^, Wageningen, The Netherlands), using a 4-day protocol. The CatWalk^TM^ system consists of a glass walkway (130 x 22 cm) that animals learn to cross from one end to the other. The walkway is enclosed by 13 cm-high plastic panels that prevent the freely moving animals from escaping and facilitate natural gait and straightforward direction. Along one side of the glass walkway, green LEDs are lined in order to highlight animals’ footprints by means of incident light. A high-speed camera positioned underneath the glass plate captures and records these illuminated areas. From day one to three, mice were trained (~30 minutes/day) to make consecutive runs over the glass runway in only one direction. Each run was rewarded by highly palatable food placed in a black box at the end of the runway. Testing was performed on day 4. For correct locomotor analysis, the following criteria concerning walkway crossing were met: i) animals had to cross the walkway without any interruption or hesitation and ii) a minimum of three correct crossings *per* animal was required [10]. Both training and test sessions were performed during the dark phase of the light/dark cycle (the active period for mice, as they are nocturnal animals), in total dark conditions (light <1 lux). Animal weight was monitored during all experiments. All data analyses were performed with a pixel threshold value ≥ 25 arbitrary units and analyzed offline. Briefly, footprints were manually classified and labelled in left front (LF), right front (RF), left hind (LH) and right hind (HR) paws. The software processed the images of labelled paws and produced a wide range of objectively calculated parameters based on the dimensions, positions, dynamics and pressure of each footfall. For each paw, one spatial and one dynamic parameter, paw angle and maximum contact at (%) respectively, was considered. Paw angle is an estimate of the angle (in degrees) of the paw axis relative to the horizontal plane. Maximum contact at (%) refers to the duration, from the start of a run, until maximum contact with glass plate occurs [1].

**Fractionation by centrifugation on glycerol gradient.** Isolated basal ganglia from WT and *Vps13a^-/-^* mice were loaded on a linear glycerol (10-40%) gradient as previously described [24,12]. The tubes were centrifuged 18 hours at 100 000*g* in a SW60Ti rotor (Beckman) at 4°C, and 18 fractions were collected from the top of each tube.

**Magnetic Resonance Spectroscopy (MRS) determination of NAA metabolite.** 1H Magnetic Resonance Spectroscopy (MRS) was performed at 7.1 T using a Bruker Avance 300 spectrometer (Bruker, Karlsruhe, Germany). Before undergoing MRI, animals were anesthetized by intramuscular injection of 5 mg/kg xylazine (Rompun; Bayer) and 20 mg/kg tiletamine/zolazepam (Zoletil 100; Virbac). T2 weighted images of the brain were acquired in the three different geometries with the following parameters: repetition time (TR) = 4000 ms; echo time (TE) = 59 ms; rare factor (RF) = 24; slice thickness = 1 mm; 15 slices; field of view = 3.00 cm; matrix = 256 × 256; number of averages (NAV) = 4; total imaging time = 2 minutes 40 seconds. A voxel of 2 x 2 x 2 mm, resulting in a volume of 8 mm^3^, was selected in the right striatum and localized shim was performed. Great care was taken to position the voxel in the same location for each animal. 1H MRS was carried out using a Point Resolved Spectroscopy (PRESS) sequence with the following parameters: TR = 2500 ms; TE = 20 ms; number of points = 2048; Spectral width = 14.98 ppm; water suppression scheme = VAPOR; number of averages NAV = 1000; total acquisition time = 41 minutes 50 seconds. Spectra were analyzed with Bruker Topspin Software: peak deconvolution was performed and the AUC underlying each peak calculated. The following metabolites were considered: N-acetylaspartate (NAA) and Creatine (Cr). As concentration of creatine is relatively constant and it is considered a most stable cerebral metabolite [2,20,23], creatine was used as internal reference to calculate metabolite ratios.

**Immunofluorescence staining for NeuN and Iba1.** Paraformaldehyde-fixed brain tissues from all mouse groups were cryostat-sectioned (Leica, section thickness 30 μm). Free-floating sections were incubated 2 hours in blocking solution at room temperature containing 2% normal goat serum and 0.2% Triton in phosphate buffer solution (PBS). Neurons were stained with NeuN antibody (Neuronal Nuclei, 1:200, Millipore) conjugated with Alexa-488 in blocking solution (5% Bovine Serum Albumin (BSA), 0.2% Triton in PBS) overnight at 4°C. Tissue slices were probed with primary antibodies against Iba1 (ionized calcium-binding adapter molecule 1, 1:500, rabbit anti-mouse antibody, Wako) in blocking solution overnight at 4°C. After rinsing 3 times with PBS 1x + 0.05% Tween, sections were processed with highly cross-absorbed goat anti-rabbit-Alexa Fluor 647 (1:500, Invitrogen, Thermo Fisher Scientific) for 1 hour at room temperature in the dark. Nuclei were stained with DAPI for 7 minutes (1:1000 in PBS 1x, Sigma).

**Immunofluorescence staining for beclin-1 and ɣ-synuclein.** Brains from *Vps13a^-/-^* mice and age- and sex-matched controls were embedded in optimal cutting temperature (OCT) compound, frozen at –80°C, and cryosectioned coronally in 35 μm thick sections. Tissue sections were blocked in 2% BSA, 2% donkey serum, 0.2% TritonX-100 in PBS 1X for 30 minutes at room temperature, followed by overnight incubation (with gentle shaking) at 4°C with anti-beclin1 (AbCam, Cambridge, UK) or anti-ɣ synuclein (ab55424, AbCam, Cambridge, UK) prepared in blocking solution. After washing in PBS 1X (10 minutes for three times), sections were incubated with the donkey anti-rabbit AlexaFluor-488 secondary antibody prepared in blocking solution for one hour at room temperature with gentle shaking.

**Caspase 3 activity.** Caspase-3 activity was determined using the CPP32/Caspase-3 Fluorometric protease assay (BioVision, Milpitas, CA, USA) following the manufacturer’s instructions.

**Proteomic analysis by nanoLC/MS-MS.** Basal ganglias samples from three different Vps13a-/-mice and from three controls (WT) were lysed by mechanical methods with pestles and by passing through syringe needle, in Bicine-HCl 25 mM pH 7.4, EDTA 1 mM, Triton 1.5%, NaF 1 mM, NaOrt 1 mM, Protease Inhibitor Cocktail Tablets “Complete mini EDTA-free”. Finally, samples were incubated for 45 minutes at 4 °C on a stirring wheel and centrifuged at 13000 rpm for 30 minutes to discard debris. Extracts protein concentrations were determined by Bradford assay. 100µg of protein extracts were separated by SDS-PAGE onto a 4-15% gradiented gel which, finally was stained with colloidal Blue Coomassie. Each lane was manually cut in 21 slices which were digested in situ with trypsin, as reported [19]. The obtained peptide mixtures were analyzed by nanoLC-MS/MS in technical duplicate by using an Orbitrap XL mass spectrometer, coupled with a nanoHPLC nanoEasy II (ThermoScientific, Waltham, MA). After loading, each peptide mixture was first concentrated and desalted onto a pre-column (C18 Easy Column L=2 cm, ID=100mm, ThermoFisher Scientific) and then fractionated on a C18 reverse-phase capillary column (C18 Easy Column L=20 cm, ID=7,5µm, 3 µm, Thermo Fisher Scientific) at a flow rate of 250 nl/min, by using a gradient from 5% to 95% of eluent B (0.2% formic acid in 95% acetonitrile) in A (0.2% formic acid and 2% acetonitrile in MilliQ water) in 80 min.

The MS/MS method was set up in a Data-Dependent Acquisition mode, with a full scan in the range from 400 to 1800 m/z, followed by fragmentation in CID modality of the top 10 ions (MS/MS scan) selected on the basis of intensity and charge state (+2, +3 charges), with a dynamic exclusion window of 40 seconds. Raw files were analysed by MaxQuant 1.5.2 integrated with Andromeda search engine for protein identification searching by using an appropriate Fasta file generated by downloading from UniProt software (2017). The selected parameters for protein identification were the following: 2 missed cleavages allowed, minimum 2 peptides required including at least 1 unique; as variable modifications were allowed methionine oxidation and pyroglutammate formation on N-terminal glutammine; accuracy for first search was set to 10 ppm, then lowered to 5 ppm in main search; 0.01 FDR was used, with a reverse database for decoy; retention time alignment and second peptides search functions were allowed. Protein quantification has been performed only using razor and unique unmodified peptides.

**MRM/MS quantitative analysis of dasatinib and nilotinib.** 50 μL mouse plasma samples were treated with 200 μL ACN/MeOH 50/50 solution to precipitate the protein fraction. The solution was centrifuged for 5 min at 12000 rpm. About 25-30 mg of mouse basal ganglia tissue (BG) were weighed. 200 μL of lysis buffer containing 7M urea, 75mM NaCl, 100mM AMBIC and 10mM NaF were added to BG samples before homogenization by T 10 basic Ultra-Turrax (IKA Dispersers). 500 μL of ACN/MeOH 50/50 solution was added to the homogenized BG samples, which were then centrifuged at 12000 rpm for 5 min. The supernatants were recovered, dried under vacuum and suspended in 200 μL of ACN/MeOH 50/50 solution to perform the MRM/MS analysis. An aliquot of 5 µL of the extracts was injected and the analysis of each sample was performed in duplicate. Quantitative analysis was performed by mass spectrometry in Multiple Reaction Monitoring (MRM) ion mode by using an AB SciexQTrap 4000 mass spectrometer, coupled with the ExpressHT™-Ultra HPLC system (Eksigent). 5 µL of the extract was injected and separated on a Halo C18 1.0 mm x 50 mm, 2.7 µm column using a 40 μL/min flow rate at 40°C during a 12 min gradient (0 min 70% A, 2min 50% A, 9min 5% A, 11 min 50% A, 12 min 70% A). The auto sampler was cooled at 4 °C. Solvent A was 1nM ammonium formate in water, acidified with 0,1% formic acid; solvent B was 1 nM ammonium formate in acetonitrile/methanol 70/30, acidified with 0.1% formic acid. Mass analysis was carried out in positive ion mode (ESI+). Precursor ion, product ions and optimal collision energies were established for each dasatinib or nilotinib MRM transition using Analyst software and are reported in Supplementary Table S2 and Table S3. The instrumental setting was CUR 20, CAD 5, IS 4500, TEMP 380, GS1 25, GS2 24. The external standard method was used to quantify dasatinib in mouse basal ganglia tissues and Nilotinib in mouse plasma samples and basal ganglia tissues. To generate a calibration curve, 2.51 mg of dasatinib standard or 0.31 mg of nilotinib standard were respectively dissolved in 1 ml MeOH/ACN 1:1 or 1 ml DMSO/MeOH/ACN 1:1:1 solution, at a range of concentrations. Dasatinib standard solutions of 10, 25, 50, 100, 250, 500, 1000, 2500 pg/μL and nilotinib standard solutions of 0.5, 1, 5, 10, 25, 50, 100, 250, 500, 1000, 2500, 5000 nM were analyzed in duplicate using the MRM/MS optimized method. According to published data [22,7,11], the *m*/*z* 488.1 → 347.2 transition or the 530,2->289.2 transition were respectively selected as quantifier, and the others were used as qualifiers. By plotting the area under the peak of the quantifier transition as a function of the concentration of the standards, it was possible to calculate the calibration curve for the target molecules.

**References**

1. Batka RJ, Brown TJ, McMillan KP, Meadows RM, Jones KJ, Haulcomb MM (2014) The need for speed in rodent locomotion analyses. Anat Rec (Hoboken) 297:1839-1864. doi:10.1002/ar.22955

2. Bertholdo D, Watcharakorn A, Castillo M (2013) Brain proton magnetic resonance spectroscopy: introduction and overview. Neuroimaging Clin N Am 23:359-380. doi:10.1016/j.nic.2012.10.002

3. De Franceschi L, Fumagalli L, Olivieri O, Corrocher R, Lowell CA, Berton G (1997) Deficiency of Src family kinases Fgr and Hck results in activation of erythrocyte K/Cl cotransport. J Clin Invest 99:220-227. doi:10.1172/JCI119150

4. De Franceschi L, Tomelleri C, Matte A, Brunati AM, Bovee-Geurts PH, Bertoldi M, Lasonder E, Tibaldi E, Danek A, Walker RH, Jung HH, Bader B, Siciliano A, Ferru E, Mohandas N, Bosman GJ (2011) Erythrocyte membrane changes of chorea-acanthocytosis are the result of altered Lyn kinase activity. Blood 118:5652-5663. doi:10.1182/blood-2011-05-355339

5. Federti E, Matté A, Ghigo A, Andolfo I, James C, Siciliano A, Leboeuf C, Janin A, Manna F, Choi SY, Iolascon A, Beneduce E, Melisi D, Kim DW, Levi S, De Franceschi L (2017) Peroxiredoxin-2 plays a pivotal role as multimodal cytoprotector in the early phase of pulmonary hypertension. Free Radic Biol Med 112:376-386. doi:10.1016/j.freeradbiomed.2017.08.004

6. Foller M, Hermann A, Gu S, Alesutan I, Qadri SM, Borst O, Schmidt EM, Schiele F, vom Hagen JM, Saft C, Schols L, Lerche H, Stournaras C, Storch A, Lang F (2012) Chorein-sensitive polymerization of cortical actin and suicidal cell death in chorea-acanthocytosis. FASEB journal : official publication of the Federation of American Societies for Experimental Biology 26:1526-1534. doi:10.1096/fj.11-198317

7. He Y, Zhou L, Gao S, Yin T, Tu Y, Rayford R, Wang X, Hu M (2018) Development and validation of a sensitive LC-MS/MS method for simultaneous determination of eight tyrosine kinase inhibitors and its application in mice pharmacokinetic studies. J Pharm Biomed Anal 148:65-72. doi:10.1016/j.jpba.2017.09.013

8. Honisch S, Gu S, Vom Hagen JM, Alkahtani S, Al Kahtane AA, Tsapara A, Hermann A, Storch A, Schols L, Lang F, Stournaras C (2015) Chorein Sensitive Arrangement of Cytoskeletal Architecture. Cellular physiology and biochemistry : international journal of experimental cellular physiology, biochemistry, and pharmacology 37:399-408. doi:10.1159/000430363

9. Kalish BT, Matte A, Andolfo I, Iolascon A, Weinberg O, Ghigo A, Cimino J, Siciliano A, Hirsch E, Federti E, Puder M, Brugnara C, De Franceschi L (2015) Dietary ω-3 fatty acids protect against vasculopathy in a transgenic mouse model of sickle cell disease. Haematologica 100:870-880. doi:10.3324/haematol.2015.124586

10. Koopmans GC, Deumens R, Honig WM, Hamers FP, Steinbusch HW, Joosten EA (2005) The assessment of locomotor function in spinal cord injured rats: the importance of objective analysis of coordination. J Neurotrauma 22:214-225. doi:10.1089/neu.2005.22.214

11. Lankheet NA, Hillebrand MJ, Rosing H, Schellens JH, Beijnen JH, Huitema AD (2013) Method development and validation for the quantification of dasatinib, erlotinib, gefitinib, imatinib, lapatinib, nilotinib, sorafenib and sunitinib in human plasma by liquid chromatography coupled with tandem mass spectrometry. Biomed Chromatogr 27:466-476. doi:10.1002/bmc.2814

12. Lupo F, Tibaldi E, Matte A, Sharma AK, Brunati AM, Alper SL, Zancanaro C, Benati D, Siciliano A, Bertoldi M, Zonta F, Storch A, Walker RH, Danek A, Bader B, Hermann A, De Franceschi L (2016) A new molecular link between defective autophagy and erythroid abnormalities in chorea-acanthocytosis. Blood 128:2976-2987. doi:10.1182/blood-2016-07-727321

13. Matte A, Bertoldi M, Mohandas N, An X, Bugatti A, Brunati AM, Rusnati M, Tibaldi E, Siciliano A, Turrini F, Perrotta S, De Franceschi L (2013) Membrane association of peroxiredoxin-2 in red cells is mediated by the N-terminal cytoplasmic domain of band 3. Free Radic Biol Med 55:27-35. doi:10.1016/j.freeradbiomed.2012.10.543

14. Matte A, De Falco L, Federti E, Cozzi A, Iolascon A, Levi S, Mohandas N, Zamo A, Bruno M, Lebouef C, Janin A, Siciliano A, Ganz T, Federico G, Carlomagno F, Mueller S, Silva I, Carbone C, Melisi D, Kim DW, Choi SY, De Franceschi L (2018) Peroxiredoxin-2: A Novel Regulator of Iron Homeostasis in Ineffective Erythropoiesis. Antioxid Redox Signal 28:1-14. doi:10.1089/ars.2017.7051

15. Matte A, De Falco L, Iolascon A, Mohandas N, An X, Siciliano A, Leboeuf C, Janin A, Bruno M, Choi SY, Kim DW, De Franceschi L (2015) The Interplay Between Peroxiredoxin-2 and Nuclear Factor-Erythroid 2 Is Important in Limiting Oxidative Mediated Dysfunction in β-Thalassemic Erythropoiesis. Antioxid Redox Signal 23:1284-1297. doi:10.1089/ars.2014.6237

16. Matte A, Low PS, Turrini F, Bertoldi M, Campanella ME, Spano D, Pantaleo A, Siciliano A, De Franceschi L (2010) Peroxiredoxin-2 expression is increased in beta-thalassemic mouse red cells but is displaced from the membrane as a marker of oxidative stress. Free Radic Biol Med 49:457-466. doi:10.1016/j.freeradbiomed.2010.05.003

17. Matte A, Pantaleo A, Ferru E, Turrini F, Bertoldi M, Lupo F, Siciliano A, Ho Zoon C, De Franceschi L (2014) The novel role of peroxiredoxin-2 in red cell membrane protein homeostasis and senescence. Free Radic Biol Med 76:80-88. doi:10.1016/j.freeradbiomed.2014.08.004

18. Matte A, Recchiuti A, Federti E, Koehl B, Mintz T, El Nemer W, Tharaux PL, Brousse V, Andolfo I, Lamolinara A, Weinberg O, Siciliano A, Norris PC, Riley IR, Iolascon A, Serhan CN, Brugnara C, De Franceschi L (2019) Resolution of sickle cell disease-associated inflammation and tissue damage with 17. Blood 133:252-265. doi:10.1182/blood-2018-07-865378

19. Medugno L, Costanzo P, Lupo A, Monti M, Florio F, Pucci P, Izzo P (2003) A novel zinc finger transcriptional repressor, ZNF224, interacts with the negative regulatory element (AldA-NRE) and inhibits gene expression. FEBS Lett 534:93-100. doi:10.1016/s0014-5793(02)03783-3

20. Minati L, Aquino D, Bruzzone MG, Erbetta A (2010) Quantitation of normal metabolite concentrations in six brain regions by in-vivoH-MR spectroscopy. J Med Phys 35:154-163. doi:10.4103/0971-6203.62128

21. Olivieri O, De Franceschi L, Bordin L, Manfredi M, Miraglia del Giudice E, Perrotta S, De Vivo M, Guarini P, Corrocher R (1997) Increased membrane protein phosphorylation and anion transport activity in chorea-acanthocytosis. Haematologica 82:648-653

22. Shen Z, Kang P, Rahavendran SV (2012) Metabolite profiling of dasatinib dosed to Wistar Han rats using automated dried blood spot collection. J Pharm Biomed Anal 67-68:92-97. doi:10.1016/j.jpba.2012.04.013

23. Stagg C, Rothman DL (2013) Magnetic resonance spectroscopy: tools for neuroscience research and emerging clinical applications. Academic Press,

24. Trentin L, Frasson M, Donella-Deana A, Frezzato F, Pagano MA, Tibaldi E, Gattazzo C, Zambello R, Semenzato G, Brunati AM (2008) Geldanamycin-induced Lyn dissociation from aberrant Hsp90-stabilized cytosolic complex is an early event in apoptotic mechanisms in B-chronic lymphocytic leukemia. Blood 112:4665-4674. doi:10.1182/blood-2008-02-139139
